# Agri-Food By-Products as Multifunctional Ingredients for Sustainable Food Oleogels: Mechanisms, Applications and Future Insights

**DOI:** 10.3390/foods15122221

**Published:** 2026-06-19

**Authors:** Giulia Salvatori, Dario Mercatante, Maria Teresa Rodriguez-Estrada

**Affiliations:** 1 Department of Agricultural and Food Sciences, Alma Mater Studiorum-University of Bologna, 40127 Bologna, Italy; giulia.salvatori5@unibo.it; 2 Interdepartmental Centre for Industrial Agrofood Research, Alma Mater Studiorum-University of Bologna, 47521 Cesena, Italy; dario.mercatante2@unibo.it

**Keywords:** fat structuring, oleogels, agri-food by-products, circular economy, upcycling, innovative food formulation

## Abstract

Agri-food by-products (BP) and BP-derived fractions are increasingly recognized as sources of functional and nutritional compounds (e.g., dietary fibers, proteins, waxes, phytosterols, phenolics, carotenoids) that can be upcycled into high-value food ingredients, to improve the sustainability of agri-food chains. This review provides a wide-ranging vision of the potential use of BP and BP-derived fractions in OG formulations, emphasizing the roles they can play (e.g., structuring agents, stabilizers, surfactants, physical scaffolds, fillers, sources of antioxidants), while offering mechanistic insights and science-based perspectives to support the rational design of tailor-made OGs for specific food applications. Particular attention is given to emerging areas including plant-based and hybrid products, and the valorization of insect BP and co-products. Finally, key gaps limiting BP-based OG design and application (e.g., effects on crystallization, interfacial phenomena, dispersion, scaffold/filler behavior, etc.) are identified and translated into a research roadmap and design guidelines for the formulation of tailor-made, scalable BP-based OGs.

## 1. Introduction

To date, more than 190 million tons of agri-food by-products (BP) are generated worldwide each year along the production chain, of which horticultural BP (such as pulp, peels, seeds, husks, pomace, pods, stems, brans and spent grains) constitute the majority [1,2]. The disposal of such huge quantities of BP contributes to the problem of food wastage and is associated with relevant economic and environmental challenges, related to the discarding of unwanted food, greenhouse gas emissions from disposal sites, the pollution of water resources, the consumption of land, and the loss of energy and water inputs used in their production [3].

At the same time, global demand for food continues to rise as the population grows, making it essential to improve the overall efficiency of food systems by preventing waste and maximizing the value recovered from the generated BP. At the European level, circular-economy strategies to reduce food waste, through the reuse, recovery, and recycling of resources, align with UN Sustainable Development Goals and promote the valorization of BP to enhance agri-food system sustainability [4,5]. Many agri-food BPs are rich in macro- and micronutrients (proteins, lipids, carbohydrates, vitamins) and bioactive compounds (polyphenols, carotenoids, dietary fiber, etc.), which can be recovered and used as ingredients or additives in food formulations, offering a concrete and valuable option to reduce food waste while improving sustainability, and nutritional and functional properties of foods [1,6,7]. Within this broader context, oleogels (OGs) represent an emerging opportunity to couple fat-structure design with BP valorization. OGs are among the most studied alternatives to conventional solid fats, which are typically rich in saturated (SFAs) and *trans* fatty acids (TFAs) [8]. By structuring liquid vegetable oils into semi-solid systems, they can mimic the technological functionality of “hard fats” while preserving a lipid profile richer in monounsaturated fatty acids (MUFAs) and polyunsaturated fatty acids (PUFAs). To date, different studies have already demonstrated the feasibility of using OGs to partially or totally replace solid fats in baked, meat and dairy products, often improving the fatty acid (FA) profile without severely compromising texture and sensory quality. However, there are still few studies exploring the potential of agri-food BPs (or their high-value compounds) for the formulation and functionalization of OGs. This approach could provide a dual benefit by supporting healthier fat systems and promoting BP valorization, thereby improving the sustainability of agri-food chains. Although several reviews have addressed edible OGs as fat replacers, bioactive delivery systems, or general food applications, the specific use of agri-food BP-derived ingredients in OG formulation remains comparatively underexplored. Previous reviews have mainly considered OG design and BP valorization as separate topics, whereas this review focuses on how BP-derived fractions can contribute to OG structure, stability and functionality. Thus, the scientific gap addressed here lies in connecting BP valorization with the rational design of sustainable food OGs.

Therefore, the aim of this review is to provide a critical overview of how agri-food BPs and their derived ingredients can be exploited for food OG design, highlighting the different roles they may play (e.g., structuring agents, stabilizers, surfactants, antioxidants, etc.) and identifying the main technological, mechanistic and regulatory challenges and gaps that limit their use in food.

### Literature Search and Screening Process

This review was designed as a descriptive narrative review with a scope-defining perspective, aimed at providing a broad and mechanistic overview of the potential use of agri-food by-products (BPs), co-products, and BP-derived fractions in food OG systems.

The literature was identified through targeted searches in Scopus, ScienceDirect, PubMed and Google Scholar, complemented by citation tracking, targeted screening of reference lists, and backward citation searching from relevant articles and official/regulatory documents when appropriate. The search strategy combined terms related to oleogelation and fat structuring (e.g., “oleogels”, “organogels”, “hard fat replacers”, non-triglyceride fat-structuring”) with terms related to by-product valorization and specific ingredient classes (e.g., “agri-food by-product”, “food by-product”, “food waste”, “co-product”, “upcycling”, “circular economy”, “waxes”, “rice bran wax”, “sunflower wax”, “phytosterol”, “lecithin”, “protein”, “dietary fiber”, “cellulose”, “saponin”, “phenolic compound”, “carotenoids”, “antioxidants”). Boolean combinations were used.

To the best of the authors’ knowledge, no previous review has specifically focused on the use of agri-food BP-derived ingredients in food OG formulation. For this reason, a two-step search strategy was adopted. Firstly, broad searches were performed to identify recent comprehensive reviews on food OGs, fat structuring, bioactive delivery, and oleogel applications, with particular attention to reviews published within the last 10 years. These reviews were used to define the main functional classes relevant to OG formulation, including structuring agents, surfactants, co-structurants, fillers and scaffolds, antioxidants, and bioactive compounds. Secondly, targeted searches were performed for each functional class by combining oleogel-related terms with ingredient-specific or source-specific terms. This approach was necessary because several compounds that have already been investigated in OG systems are not always discussed as BP-derived ingredients, even though they may originate from agri-food side streams or co-products. Examples include vegetable waxes and phytosterols recovered from vegetable oil-refining streams, lecithin from the degumming step, and protein fractions obtained from dairy or oilseed processing co-products. Therefore, the literature was screened not only for studies explicitly addressing BP-based OGs, but also for studies providing mechanistic insights or functional information on ingredients that may be obtained from BPs or co-products.

As an illustrative example, the direct query “oleogels AND food by-products” retrieved 14 records in Scopus, confirming that BP-based OGs are still poorly represented as a unified research topic. Additional searches were therefore conducted using combinations such as “oleogels AND plant fibers”, “oleogels AND ethylcellulose”, “oleogels AND waxes”, “oleogels AND phytosterols”, “oleogels AND lecithin”, “oleogels AND proteins”, and “oleogels AND phenolic compounds”. This also allowed us to distinguish between well-studied model or purified systems, such as ethylcellulose-, hydroxypropyl methylcellulose-, or microcrystalline cellulose-based oleogels, and more heterogeneous, less-explored BP-derived fractions, such as minimally processed plant fibers or complex lignocellulosic residues, including citrus fibers, bamboo fibers, tomato fibers, and brewer’s spent grain.

Peer-reviewed articles and relevant official documents were included when they addressed: (i) formulation, characterization or application of food-grade OGs or OG-based systems, with and without BP inclusion; (ii) ingredients, fractions or bioactive compounds obtained from agri-food BPs or co-products; (iii) structuring, stabilizing, surfactant, filler/scaffold, antioxidant or bioactive-delivery roles of such compounds (inside and outside OG systems); and/or (iv) food applications related to fat replacement, bioactive delivery or sustainable formulation. Studies were excluded when they were unrelated to food systems or focused exclusively on non-food pharmaceutical or cosmetic applications without transferable relevance; papers that lacked sufficient methodological or compositional information, or fell outside the thematic scope of the review, were not considered as well.

Overall, the final reference list included 209 sources, comprising review articles, original research articles, book chapters, and relevant official/regulatory documents. The sources included publications from major scientific publishers in food science, lipid chemistry, colloid science, and food technology, as well as official documents from regulatory authorities. Priority was given to publications from the last 5–10 years, reflecting the rapid development of research on food OGs and by-product valorization. However, no strict publication date cut-off was applied, and earlier studies were retained when they provided relevant information on oleogelation mechanisms, ingredient functionality, regulatory aspects or bioactive compounds. Accordingly, the majority (>80%) of references included in the review were published from 2016 onwards, as reported in the final reference list.

## 2. Oleogels

### What Are Oleogels and Their Main Advantages?

**Figure 1:**
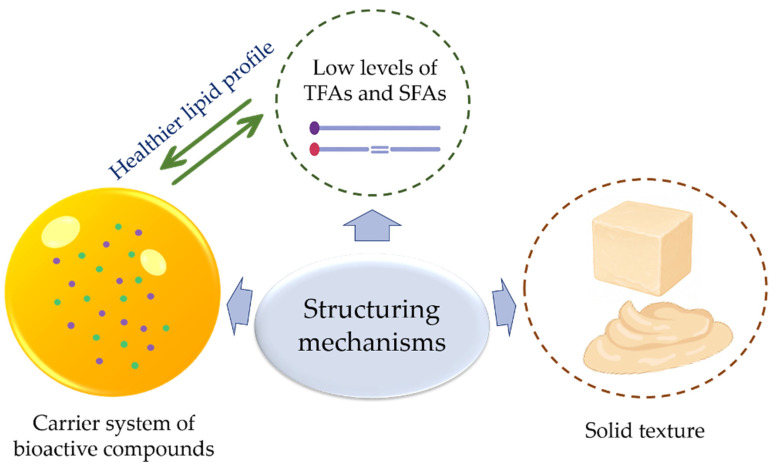
Potential benefits of oleogels.

Gels represent colloidal systems with the mechanical properties of solids, consisting of at least two components, such as a continuous liquid phase entrapped inside a three-dimensional (3D) network [9]. Oleogels (OGs) are oil-based gels in which edible oils are entrapped in a thermo-reversible 3D network formed by non-triglyceride structuring agents, known as oleogelators [10]. In particular, a wide range of gelators have shown the ability to form 3D network structures at concentrations below 15%, through non-covalent interactions (e.g., hydrogen bonding, electrostatic and van der Waals forces), leading to the production of solid-like systems with viscoelastic properties [11,12]. Nowadays, OGs represent one of the promising alternatives for replacing solid fats (also called “hard fats”), which are usually rich in SFAs and TFAs [13]. Since these FA classes are crucial for food texture and flavor [14], there is a need for solutions that preserve the functional and sensory attributes of solid fats while improving the lipid profile and overall sustainability. Unlike hydrogenation or interesterification, oleogelation does not change the chemical composition of the starting oil, allowing the nutritional profile rich in unsaturated fatty acids (UFAs) to be preserved while providing the technological and sensory functions of solid fat (Figure 1). For this reason, many studies have included OGs in the formulation of various food products (such as baked, meat, dairy and confectionary goods) [15]. Furthermore, OGs have been evaluated as effective carrier systems for bioactive compounds in food products [16]. Many bioactive compounds, especially highly hydrophobic ones, show poor solubility and dispersion in food matrices [17], and therefore require encapsulation in colloidal delivery systems to ensure stability and bioavailability during processing and storage [18,19,20]. OGs may represent an alternative vehicle to improve solubility, dispersibility, release modulation, and protection against oxidation; however, most studies focus on fat-soluble compounds, while *in vivo* evidence on digestion, release, and absorption remains limited [9].

In this context, agri-food BPs represent promising sources of both structuring agents and bioactives that can be integrated into OG systems.

## 3. Exploring Agri-Food By-Products in the Formulation of Oleogels

**Figure 2:**
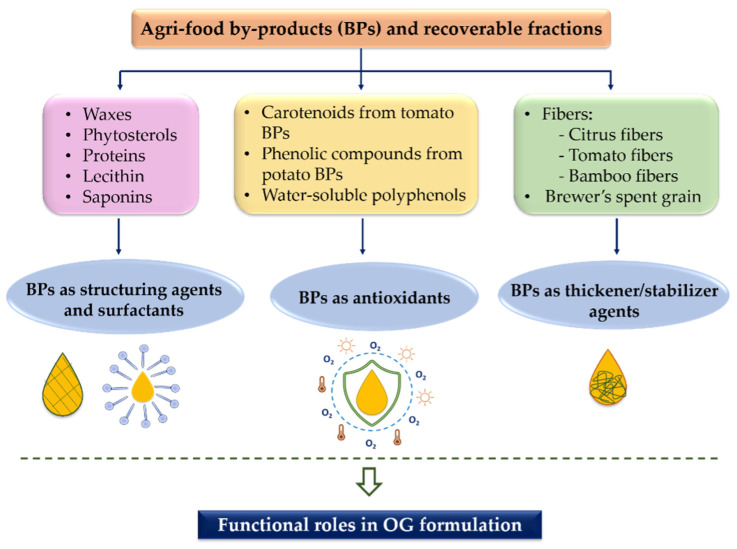
The main agri-food BPs and recoverable fractions described in the present review.

Agri-food BPs (such as pulp, peels, seeds, husks, pomace, pods, stems, brans and spent grains) are rich in compounds such as polyphenols, carotenoids, β-glucans, dietary fibers and proteins, and can be valorized as functional food ingredients [7]. Given these multiple functionalities, BP-derived compounds can be used, alone or in combination, to tailor OG properties. As mentioned above, OGs can also act as carrier systems of bioactive compounds, thus enhancing their dispersion, protecting them from oxidation, and modulating their release and bioavailability [9,18,19]. Therefore, in line with the principles of the circular economy, upcycling, and sustainable development, OGs represent an attractive strategy for the utilization and valorization of such BPs. However, BP-derived components should be carefully evaluated because they may interfere with network formation and alter the mechanical or thermal properties of OGs. In the next paragraphs, the main components that can be recovered from some agri-food BPs will be described according to the roles they can play in OG formulations, as schematically illustrated in Figure 2.

In this review, BP-derived ingredients are considered at different levels of processing and market readiness. Genuine by-products or residues, such as peels, pomace, brans, brewer’s spent grain, and olive mill by-products, are often underutilized streams requiring stabilization, extraction, or functionalization. In contrast, other by- and co-products (such as whey or defatted oilseed meals) already have established uses and economic value, but can still represent relevant sources of functional fractions for higher-value food applications. Finally, whey protein isolates (WPIs), soy protein isolates (SPIs), lecithin (LC), rice bran wax (RBW), and phytosterols (PSs) may originate from by-products or refining side streams but are already available as standardized commercial ingredients.

Accordingly, the innovation discussed in this review may lie either in the value-added utilization of underexploited BPs, in the incorporation of commercial BP-derived ingredients into OG formulations, or in both, depending on the ingredient considered. For already commercial ingredients, the novelty lies primarily in their use as functional components for designing sustainable food OGs, rather than in their recovery or production process. For less exploited residues, such as brewer’s spent grain (BSG), tomato peels, olive mill by-products, or insect-derived co-products, both the valorization route and the OG formulation strategy remain relevant research opportunities.

The following sections discuss the main BP-derived ingredient classes according to their dominant functional roles in OG systems, while a cross-sectional comparison of their mechanisms, technological performance, and application constraints is provided in Section 3.4.

### 3.1. By-Products as Structuring Agents and Surfactants

Many agri-food BPs contain several components such as vegetable waxes, PS, LC, proteins, etc., which can be successfully employed as structuring agents and surfactants in OG formulations.

An ideal structuring agent should be food-grade, cost-effective, versatile, and able to structure oils without impairing sensory quality. However, it is rather challenging to simultaneously meet all these criteria [21]. Oleogelators are commonly grouped into low-molecular-weight (LMW) and high-molecular-weight (HMW) classes [15] (Table 1), and the main oleogelators used in food systems, their BP sources, and typical structuring conditions are summarized in Table 2.

**Figure 3:**
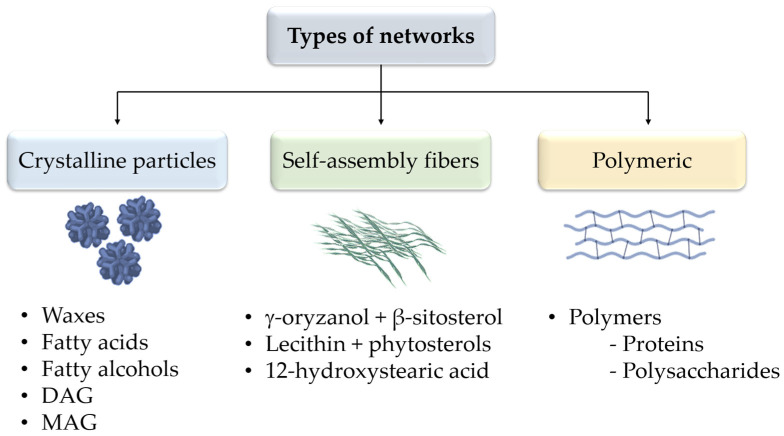
Types of networks formed with the most commonly used gelling agents.

Depending on gelator properties, three main types of networks can be distinguished (schematically illustrated in Figure 3), namely crystalline, self-assembled and polymeric networks. The first type arises from nucleation, crystal growth and aggregation, and it is typically observed in OGs structured with waxes, MAG, FA, and FAOH [22,23]. Self-assembled networks derive from the organization of molecules into elongated structures such as tubules or fibrils that entangle, forming a fibrillar network, as reported for β-sitosterol/γ-oryzanol blends, 12-hydroxystearic acid, and LC in the presence of small amounts of water [24,25]. Polymeric networks, in turn, are generated with polymeric gelators.

In the production of OGs, the kinetics of network formation and the resulting macro- and microstructural properties depend not only on the chemical nature and concentration of the gelator, but also on oil composition, polarity, degree of unsaturation, carbon chain length, minor components, and gelator–oil affinity. These variables can markedly affect crystallization and self-assembly, meaning that the same oleogelator may develop OGs with different physico-chemical characteristics depending on the oil system used [26].

**Figure 4:**
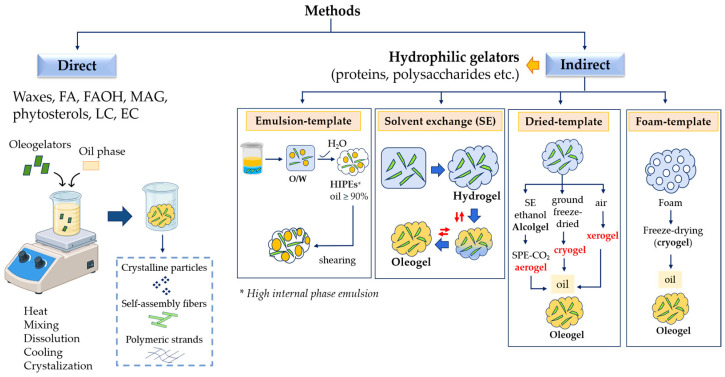
Overview of OG structuring routes: Direct and indirect approaches.

**Figure 5:**
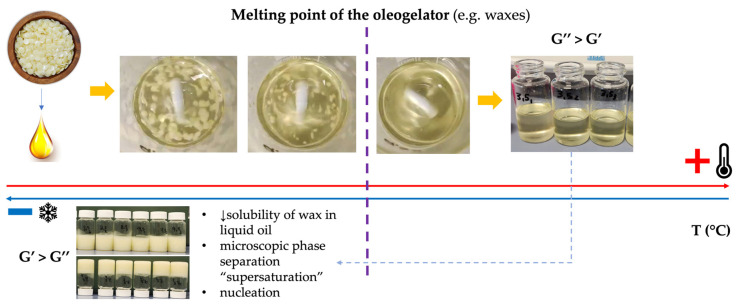
Brief scheme of the oleogelation mechanism via direct gelator dispersion.

From a processing standpoint, these systems can be produced by direct or indirect approaches (Figure 4). In the direct methods (Figure 5), gelators are dispersed in oil, heated above their melting point, and then cooled to create crystalline or self-assembled networks. So far, EC is the only known polysaccharide that can be used to form OGs through a direct dispersion approach [27]. Indirect approaches include solvent exchange and emulsion-, foam- or dried-template routes. In these approaches, a hydrogel, emulsion, or porous dry matrix is first prepared in an aqueous environment; water is then removed and replaced with oil, thus enabling the use of hydrophilic biopolymers that are generally unsuitable for direct dispersion in lipid systems [28,29,30,31]. From a sustainability and scalability perspective, however, the oleogelation route should also be considered when BP-derived ingredients are used. A recent quantitative analysis of 216 oleogelation cases identified three industrially relevant parameters for comparing OG production methods: the total heat applied to the oil, electrical energy consumption, and overall processing time. This analysis showed that low-input methods are generally the most promising in terms of oxidative stability, sustainability, and industrial relevance, but also that direct methods are not necessarily superior to indirect methods in all cases; therefore, oleogelation routes should be evaluated on a case-by-case basis [32]. In this context, direct approaches (one-step) may be more easily transferable when liposoluble BP-derived ingredients are used, whereas indirect approaches expand the range of usable hydrophilic BPs or BP-derived fractions, such as proteins and polysaccharides, but may require additional energy, water removal, drying, solvent exchange, or solvent recovery steps. In particular, dried-template and solvent exchange routes may increase energy demand, processing time, and solvent-related constraints, while emulsion-template and semi-direct capillary-suspension approaches may be more promising for scale-up when they rely on food-compatible operations and commercially available ingredients or particles [32,33]. Therefore, the sustainability of BP-based OGs should not be assumed solely on the basis of BP valorization, but should be verified considering processing inputs, solvent and water use, drying requirements and ingredient availability, together with the logistics of BP collection, stabilization, storage, and transport. Future studies should actually integrate life cycle assessment (LCA) and techno-economic evaluation to confirm the sustainability of BP-based OGs at pilot and industrial scales.

More recently, capillary-suspension oleogelation has emerged as a non-thermal structuring strategy whereby particles dispersed in oil become interconnected through capillary bridges formed upon the addition of small amounts of an immiscible secondary liquid (typically water), leading to the formation of rigid, gel-like structures [34]. This approach does not involve the dissolution of a structuring agent in the oil, but the prior formation of capillary suspensions, whose strength mainly depends on particle fraction, size and secondary-fluid content [35,36]. So far, starch granules, cellulose particles, agri-food residues and BPs (e.g., tomato peels, coffee grounds, soybean protein isolate, and protein particles/aggregates (e.g., zein and whey protein isolate)) have been used to form capillary suspensions [37,38,39,40]. This strategy appears promising for incorporating plant fibers and BP-derived particles into OG systems, even though particle heterogeneity and the need for pre-treatments (e.g., high-shear mixing, high-pressure homogenization, sonication) to modulate granulometry and surface properties, still limit its broader application [36].

This mechanistic and processing diversity is essential to interpreting the potential of the BP-derived compounds discussed below, since each class of ingredient contributes to OG formation through distinct pathways and formulation constraints. Among these, waxes currently represent the most established and efficient examples.

**Table 2:** Overview of oleogelators obtained from agri-food by-products: Their sources, structuring methods, oil systems, and key processing parameters.

Gelators (Structuring Agents)	Main Sources of Gelators	Liquid Phase	Structuring Mechanism	Ref.
Sunflower wax (SFW)	Obtained by winterization of sunflower (*Helianthus annus*) oil during refining	Soybean oil	Direct dispersion approach: - Addition of 5% SFW in oil; - Heating at 70–80 °C to dissolve SFW; - Cooling period at room temperature.	[41]
		Soybean oil Olive oil Flaxseed oil	Direct dispersion approach: - Addition of 8% SFW in oil; - Heating at 80–90 °C to dissolve SFW; - Cooling period at room temperature.	[42]
Sorghum wax (SGW)	Extraction from sorghum (*Sorghum vulgare* Pers.) kernel and/or bran	Fish oil	Direct dispersion approach: - Addition of 2, 4, 6, 8, and 10 wt% of SGW in oil; - Heating at 85 °C to dissolve SGW; - Ultrasonic treatment and cooling period at different cooling rate.	[43]
Rice bran wax (RBW)	Obtained by winterization of rice bran (*Oryza sativa*) oil during refining	Rice bran oil	Direct dispersion approach: - Addition of 0.5, 1, 3, 5, 7, 10, 15, 20, and 25 wt% of RBW in oil; - Heating at 90 °C to dissolve RBW under continuous stirring conditions (250 rpm using a magnetic stirrer); - Cooling period at room temperature.	[44]
Candelilla wax (CDW)	Obtained from the leaves of Candelilla shrubs (*Euphorbia cerifera*) Authorized by the EU as coating agents under the food additives European Regulation No. 1333/2008 [45], with the code E902	Mixture of rapeseed oil and linseed oil	Direct dispersion approach: - Addition of 30–80 g/kg of CDW in a mixture of oils (1:1); - Heating at 80 °C in water bath for 10 min and sonication for 20 s; - Cooling period at room temperature.	[46]
Carnauba wax (CRW)	Obtained from palm tree leaves (*Copernicia cerifera*) Authorized by the EU as coating agents under the food additives European Regulation No. 1333/2008 [45], with the code E902	Soybean oil	Direct dispersion approach: - Addition of 5, 10, 15 wt% of CRW in oil; - Heating at 80, 90 and 100 °C for 30 min in an agitated water bath; - Different cooling temperatures (5, 10, and 15 °C) for 24 h.	[47]
Beeswax (BW)	Produced by bees during the formation of hives Authorized by the EU as coating agents under the food additives European Regulation No. 1333/2008 [45], with the code E901	Camellia oil Soybean oil Sunflower oil Flaxseed oil	Direct dispersion approach: - Addition of BW in different oils; - Heating at 85 °C in a water bath; - Cooling period at room temperature overnight after mixing thoroughly. Each prepared sample was placed in a 60 mL glass bottle at room temperature (25 °C) to investigate the critical concentration by inverting the samples after 24 h	[48]
Lecithin (LC)	Obtained from the degumming step of vegetable oil refining (commonly from soybean and sunflower oils)	Canola oil	Direct dispersion approach: - Addition of lecithin to oil; - Mixing overnight at 60 °C until the LC was dissolved and the contents were visually homogeneous; - Addition of water dropwise with a micropipette and the contents mixed until homogeneous; - Samples were left at ambient temperature and gelation took place within 1–12 h depending on the formulation.	[49]
Soybean lecithin (SLC) (and ethylcellulose (EC))	Obtained from the degumming step of soybean oil refining	High-oleic canola oil	Direct dispersion approach: - A first solution was made by dispersing 10, 11 or 12 wt% of EC into 95% of total mass fraction of the oil and heating at 140 °C for 40 min; - A second solution was made by dispersing 1, 3 or 5 wt% of LC into 5% of total mass fraction of oil, and heating at the same temperature for 5 min; - Both solutions were mixed and heated for approximately 5 min until complete dissolution was observed; - Heating and mixing of the OGs at a constant rate (∼175 rpm) were achieved with a bench-top gravity convection oven coupled to an external overhead mechanical stirrer; - Cooling and storing at room temperature for 24 h before analysis. In addition, samples obtained by simultaneously mixing EC and SLC were also produced. In this case, fractionated and fully hydrogenated SLC with a phosphatidylcholine content of 90% was used.	[50]
Sitosterol (and LC)	Plant sterols can be isolated from vegetable oil-refining side streams (such as deodorizer distillates) or from crude tall oil (a major by-product of cellulose production) by fractional distillation	Sunflower oil	Direct dispersion approach: - Addition of 16% (wt%, based on oil) of sitosterol and LC in oil at a temperature of 90 °C for 40 min, under continuous stirring; - Sitosterol: LC mass ratios were: 0:10, 6:4, 7:3, 8:2 and 10:0 at 16% total structurant concentration; - Cooling period and storing overnight at 5 °C.	[51]
β-sitosterol (BS) (and γ-oryzanol)	Plant sterols can be isolated from vegetable oil-refining side streams (such as deodorizer distillates) or from crude tall oil (a major by-product of cellulose production) by fractional distillation	Sunflower oil	Direct dispersion approach: - Addition of BS and γ-oryzanol at a mass ratio of 4:6 in oil; - Heating at 90 °C and stirred until the sterols had fully dissolved; - Cooling at room temperature.	[52]
Whey protein isolate (WPI)	Extracted from whey, a by-product of cheese manufacturing	Demineralized water Sunflower oil	Solvent exchange approach: (a) Preparation of protein aggregates: - WPI powder (4 wt%) was dissolved in demineralized water under continuous stirring at room temperature for 2 h. The solution was stored overnight at 4 °C to assure complete protein hydration. - pH adjustment of the stock solution with 1 M HCl. - Heating the solution at 85 °C for 15 min to induce protein denaturation. - Cooling in ice water to obtain a weak protein gel (hydrogel). - Homogenization of the dispersion at 13.000 rpm for 3 min. - Collection of protein aggregates as a pellet by centrifugation. (b) Preparation of the protein oleogels: - WPI aggregates were added to oil through a solvent exchange procedure by using acetone as an intermediate solvent; - The polarity of the solvent was changed gradually to remove water and replace it with oil as continuous phase.	[53]
Soybean protein isolate (SPI)	Obtained from defatted soybean meal/flakes, a co-product/side stream of soybean oil processing	Sunflower oil Distilled water	Emulsion-template approach: - Preparation of an aqueous SPI or SPI:κ-carrageenan dispersion; - Homogenization with oil to form an emulsion (60% oil/40% aqueous phase); - Drying of the emulsions to obtain oleogels.	[54]

Footnotes: BS: β-sitosterol; BW: beeswax; CDW: candelilla wax; CRW: carnauba wax; LC: lecithin; RBW: rice bran wax; SFW: sunflower wax; SGW: sorghum wax; SLC: soybean lecithin; SPI: soybean protein isolate; WPI: whey protein isolate.

#### 3.1.1. Waxes

Waxes are organic hydrophobic heterogeneous mixtures of long-chain FA esterified with FAOH, characterized by low polarities and high melting points. The long alkyl chains may include different functional groups (e.g., esters, ketones, aldehydes, hydroxyls) and present unsaturated bonds [11]. The major constituents are wax esters (WEs), free fatty acids (FFAs), FAOHs, hydrocarbons (HCs) and other minor components (<10%) [55].

The distribution of WEs or *n*-alkanes into small microcrystals that subsequently self-aggregate is believed to be the fundamental mechanism driving wax gelation [56]. Because of their multicomponent nature, waxes exhibit complex gelling behavior that is largely determined by their chemical composition, including the alkyl chain length of WE, FFA, and FAOH, their relative proportions, and degree of unsaturation. These factors jointly influence the physico-chemical properties of waxes, such as melting point, solubility, and critical gelling concentration (CGC) [55]. In particular, the chain length of WE influences the melting point and CGC of waxes. A compositional profiling study conducted by Doan et al. [57] showed that plant waxes differ markedly in the relative proportions of WE, FAOH, HC and FA. In agreement with these findings, Wettlaufer et al. [58] demonstrated that compositional differences among waxes strongly influence their gelation behavior in oils. Waxes rich in WE (such as sunflower wax (SFW) and rice bran wax (RBW)), in HC (such as beeswax (BW) and candelilla wax (CDW)), or in FAOH (such as carnauba wax (CRW) and sugarcane wax) give rise to markedly different OG structures and properties. The melting points of some of the main waxes used for OG formulation are summarized in Table 3.

##### Sources

In the agri-food chain, relevant wax sources include oil-refining streams (e.g., RBW, SFW), leaves of specific plants (CRW, CDW), and cereal kernel/bran fractions (e.g., SGW). During the extraction process of vegetable oil, a lot of minor components (phospholipids, waxes, tocopherols, FFAs) are extracted together with the oil and must be removed because of their negative impact on the quality and stability of oils [62]. In the case of waxes, they are undesirable components due to their tendency to crystallize at room temperature, which leads to turbid or “veiled” oils. The removal of these compounds can be performed during the refining process through different methodologies. The most common approach is the winterization process (de-waxing), which exploits their tendency to crystallize for separating them from other components. The process comprises an initial cooling of the oil by agitating it mechanically to cause wax crystallization. Thereafter, the crystals are separated from the oil by filtration using filters and filter aids (e.g., diatomaceous earth), generating “filter cakes”, which represent an exploitable refining side stream for wax recovery [62].

##### Application in OGs

Due to their highly hydrophobic nature, waxes are widely used in the food industry as edible coatings to extend shelf-life by reducing moisture loss and limiting gas exchange [63]. BW, CDW, and CRW are approved as food coating agents in the EU (E901, E902, and E903, respectively) and can form crystalline networks, making them suitable structuring agents for OGs produced through direct-dispersion approaches. Waxes are considered among the most promising gelators because they combine high OBC at low concentrations, wide availability, affordability, and food-grade status [11,64]. After being dissolved within an organic liquid phase above their specific melting point, waxes crystallize upon a cooling phase, forming crystalline self-sufficient networks which entrap the liquid oil within a solid-like structure [58]. The characteristics of OGs, such as strength, consistency, and stability, can be modulated by wax type and concentration, solvent composition, cooling rate, and shear conditions during preparation, as these factors influence gelator–solvent interactions and ultimately determine the resulting gelling properties [65]. The structuring capacity that waxes display is mostly ascribed to the presence of long-chain WE, but also depends on their concentration, the overall composition of the wax, the cooling rate, the shear applied, and the type of solvent [58]. To date, there have been many studies in which waxes have been used as gelling agents to create OGs (see Table 2), and which aimed at understanding the main parameters for modulating gelator–solvent interactions. The most extensively studied waxes include CDW, CRW, BW, and SFW, but RBW and sorghum wax (SGW) have recently gained interest.

Hwang et al. [41] showed that SFW esters form plate-like crystals whose morphology and gelling efficiency depend on oil type. In a subsequent study, Hwang et al. [42] evaluated the performance of OGs made with various oils (olive, soybean, linseed) and waxes (SFW, RBW, BW, and CDW), to be used as margarine replacers in cookies. SFW and RBW generated the firmest doughs, while cookies made with OGs showed similar properties to those prepared with commercial margarine, thus supporting their viability in baked applications.

Thakur et al. [47] conducted a very interesting study aimed at optimizing soybean oil OGs structured with CRW to be used as frying media, by applying response surface methodology to evaluate wax concentration and heating and cooling temperatures. The optimal formulation (8% CRW, 92 °C heating, 10.7 °C cooling) showed high oil-binding capacity (97.31%), a higher smoke point (283 ± 2 °C) than soybean oil (232 ± 0.4 °C), and reduced FFA content, thus indicating its suitability as a frying medium.

Szymańska et al. [46] compared palm oil with CDW-based OGs (30–80 g/kg) produced from rapeseed/linseed oil blends (1:1). CDW concentration was able to significantly alter physical parameters, such as color, crystal size and morphology. Increasing CDW content reduced structuring time and improved gelation temperature, elasticity, and physical stability, with OGs showing higher centrifugal resistance than palm oil, even at the lowest concentration tested. CDW formed grain-like crystals similar to palm oil but with a smaller average crystal size, supporting its potential as a palm oil replacer.

On the other hand, Liu et al. [43] investigated the use of *Sorghum vulgare* Pers as a promising alternative natural wax source. These authors were the first to evaluate the oleogelation potential of three sorghum-derived waxes (sorghum bran wax (SBW), distillers’ dried grains and solubles wax (SDW), and sorghum kernel wax (SKW)) as natural gelators for fish oil, thereby demonstrating a promising route for the valorization of sorghum by-products. All waxes produced stable OGs, with SDW showing the greatest oxidative stability. Additionally, fast cooling and ultrasound improved gelling performance by promoting smaller crystal formation, thus reducing oil loss. The authors ascribed this outcome to the capacity of these two processing parameters to result in an earlier onset of crystallization and a shorter melting interval.

Over the past few years, RBW has also gained attention for its high efficiency as a gelator. Wijarnprecha et al. [44] showed that RBW can gel vegetable oil at concentrations as low as 0.5 wt% and that its high-aspect-ratio crystals contribute to strong structuring ability across a broad concentration range (0.5–25 wt%).

Finally, Wettlaufer et al. [58] demonstrated the crucial role of the liquid phase in the production of wax-based OGs by comparing OGs produced with canola oil (rich in MUFAs, especially C18:1 *cis*-9) and medium-chained triacylglycerol (MCT) oil (composed of C8:0 and C10:0 FA), using six different waxes. This choice allowed assessment of the effects of a common oil (with typical minor components and UFAs) and a “synthetic” oil (without minor components and with only SFAs). The study revealed that both wax and oil composition significantly influence crystallization behavior, gelling process and final OG properties, highlighting the need to consider solvent quality alongside wax type and concentration.

#### 3.1.2. Phytosterols

Plant sterols (or phytosterols, PSs) are high-value compounds with antioxidant properties and cholesterol-lowering effects. These compounds have attracted considerable interest because of their potential health benefits, particularly their ability to reduce the risk of cardiovascular diseases by lowering total cholesterol and low-density lipoprotein (LDL) cholesterol levels when consumed at daily intakes of 2.5–3 g [66,67]. As a consequence of their beneficial effects on human health, many food products, especially dairy products, have been enriched with these bioactive compounds, considering the health claim regulations issued by the FDA [68] and the European Commission [69] following a positive opinion by EFSA.

##### Sources

PSs are present in all plant foods, particularly in oilseeds and in vegetable oils. Whole grains, fruits and legumes are also important dietary sources of PSs [67,70]. However, vegetable oils are usually subjected to refining, where the loss of PSs during the deodorization phase can exceed 40% [71]. Therefore, refining BPs, especially deodorization distillates (DDs), represent a rich source of PSs and are commonly used for the recovery of these compounds [72]. Hence, the recovery of PSs from the BPs of vegetable oil refining represents a possible way of valorizing these BPs for their incorporation into value-added food products.

##### Application in OGs

In recent years, PSs have attracted growing interest as oleogelators, because they can structure edible oils mainly through synergy between the individual components (co-assembly) [8,73]. In particular, blends of β-sitosterol and γ-oryzanol (sterol ester) form translucent OGs by co-assembling into elongated tubules that entangle into a fibrillar network [8]. γ-oryzanol is a combination of PSs and various triterpene alcohol esters of ferulates (4-hydroxy-3-methoxycinnamic acid) extracted from rice bran oil, which exhibits antioxidant activity ascribable to its ferulic group [74].

The strength and morphology of these networks depend on the oil environment, as oil polarity and minor polar components can modulate sterol–sterol interactions and, consequently, the thermal and mechanical properties of the gels. Sawalha et al. [75] demonstrated that oil polarity strongly affects the self-assembly of PS networks. Oils with low dielectric constants produced curly, banded tubules, whereas higher-polarity oils (e.g., castor, sunflower) yielded straighter and more densely packed tubules associated with stronger gels. At very high polarity, single tubules also appeared. Moreover, plant oils contain minor polar components that can influence assembly, as they can interact with the ferulic groups of γ-oryzanol, enhancing intermolecular interactions at low concentrations and thus increasing gel hardness [76]. Overall, oil polarity, unsaturation level, and minor polar compounds are key parameters governing tubule morphology and the mechanical strength of PS-based OGs.

From a formulation perspective, the physical state of PSs remains a critical issue in OG systems. Pure PSs have limited solubility in both oil (3–4%) and water (1–2%) [77] and readily crystallize at higher concentrations, which restricts their incorporation into food products and may reduce their biological efficacy. Indeed, PSs must be present in a soluble rather than crystalline state to effectively exert their cholesterol-lowering activity [78]. Moreover, the crystal size of PSs influences their bioactivity: the smaller the size, the larger the total surface area and the more extensive the competitive effect on the (re)absorption of cholesterol [79]. Therefore, strategies such as emulsification of PS-oryzanol OGs have been proposed to limit sterol crystallization and promote higher apparent solubility, supporting the design of more stable PS-enriched systems.

Moschakis et al. [78] prepared OG-in-water emulsions (20% OGs based on γ-oryzanol and PS) and incorporated them into a model yogurt to investigate whether emulsification could improve the physical state of PSs by reducing crystal size and limiting crystallization. This approach was expected to enhance their physiological functionality in the intestinal environment while also influencing the rheological properties of the final product. Emulsification increased PS solubility in oil, inhibited crystallization up to 15 wt% PS concentration (vs. the usual 3 wt% in oil), and enhanced emulsion stability, suggesting improved potential bioactivity. PS crystallization was found to be dependent on both the γ-oryzanol:PS ratio and total PS content, with OGs prepared at equivalent molar ratios remaining solid and stable after cooling. These results suggest that emulsification of PS-oryzanol OGs may represent a promising strategy for designing functional, PS-enriched foods with enhanced bioactivity.

Matheson et al. [52] showed that increasing sterol–oryzanol concentration enhances fibril branching and microstructural dimensionality, thereby influencing macroscopic rheology. Although gels arise from one-dimensional fibrils, their aggregation into ribbon-like, branched networks appears central to the structure and mechanical behavior of PS-based OGs. The authors therefore suggest that fibril–fibril interactions may be crucial to understanding overall structure formation and network development in this system.

Overall, PS-based OGs represent a promising platform for combining lipid structuring with potential health benefits, even though their performance remains strongly dependent on oil composition and formulation conditions.

#### 3.1.3. Proteins

Among the available oleogelators that have been used to structure liquid oils, proteins are among the most recently investigated candidates and have attracted considerable interest due to their nutritional value and high level of consumer acceptance [80].

##### Sources

Among agri-food BPs, proteins can be obtained from cereal and legume supply chains. Generally, protein concentrates and isolates of vegetable origin are recovered from BPs of vegetable oil extraction processes (as cakes or “flours”). Soybean is a key example, since the production of soybean oil yields large amounts of defatted soybean meal (≈44–48% protein), which can be considered a valuable co-product rather than a simple BP. Indeed, in this regard, soybean represents an exception among oilseeds, considering that for every ton of raw soybean oil produced, more than 4 tons of soybean meal are generated [81]. Considering soybean protein production as an example, Preece et al. [82] explained in detail that to produce protein concentrates and isolates, hulled soybeans are usually conditioned to prepare soybean flakes with optimal thickness to facilitate solvent extraction of the oil. After removal of the oil, defatted soybean flakes (or defatted soybean meal) can be used as feed or to extract protein as a higher-value route. To produce soybean protein concentrate (SPC), the defatted soybean flakes undergo a series of processing steps to remove unwanted components, such as carbohydrates, by adding alcohol or water. Soybean protein isolate (SPI) contains a higher protein content than SPC, as it undergoes further purification steps to also remove insoluble carbohydrates and dietary fiber through an intermediate acid precipitation step.

##### Application in OGs

Indirect dispersion methods have enabled the use of biopolymeric oleogelators, including proteins and polysaccharides. However, most OGs and OG-based systems (e.g., bigels and emulsions) still rely on animal-derived proteins (such as whey proteins, caseinates, gelatin, and egg white proteins), whereas plant proteins have been comparatively less investigated [83]. Their generally lower emulsifying capacity, solubility, water-holding capacity (WHC), and gelling or foaming properties often limit the technological performance of plant proteins. Some researchers have developed OGs through different indirect methodologies, using SPI, legumes and canola protein isolates (CPIs).

Tavernier et al. [54] produced OGs via an emulsion-templated approach using SPI and SPI:κ-carrageenan (κ-CG) to investigate whether hydrophilic proteins and protein:polysaccharide complexes were efficient structuring agents. A water dispersion of SPI and the SPI:κ-CG complex was first prepared; afterwards, an aqueous emulsion was made by dispersing 60% oil in a 40% aqueous dispersion of SPI:κ-CG or SPI using a homogenizer. The emulsions were then dried to form OGs, revealing that SPI alone produced stronger OGs, whereas SPI:κ-CG complexes improved long-term stability due to increased viscosity and accumulation at the interface of the protein:polysaccharide complexes.

Mohanan et al. [84] were among the first to apply a foam-templated approach to legume proteins for OG production. Pea and fava bean protein concentrates and isolates were combined with xanthan gum (XG) across a range of pH values, whipped into foams, freeze-dried, and used to structure canola oil. Stable foams were obtained with 5% protein and 0.25% XG at pH 3–9. Protein concentrates yielded OGs with higher OBC, resulting in smaller pores of freeze-dried foams, but lower gel strength than isolates; these outputs were consistent with the higher final oil content. Among concentrates, foams prepared at pH 5 showed poor stability and collapsed during oil incorporation, resulting in lower OBC. Overall, the study demonstrated that legume proteins, particularly in combination with XG, can effectively structure OGs and further highlighted the role of protein–polysaccharide interactions within freeze-dried foams in determining OBC and the rheological and structural properties of the resulting gels.

Tang and Ghosh [85] demonstrated that heat-induced denaturation of canola protein isolate (CPI) enhances OBC and rheological performance in emulsion-templated OGs. Emulsions of canola oil (50 wt%) stabilized with 1–4 wt% CPI were homogenized, heated (90 °C, 30 min), dried, and sheared to form OGs. Heated emulsions containing 4 wt% CPI produced OGs with minimal oil loss and the highest gel strength, viscosity, and firmness, enabling full replacement of shortening in cake formulations. Cakes prepared with these OGs showed greater specific volume, larger air channels stabilized by CPI aggregates, reduced hardness, cohesiveness, and elasticity compared with shortening-based controls.

#### 3.1.4. Lecithin

Lecithin (LC) represents a complex mixture of phospholipids, glycolipids and TAG [86], which can be obtained from both vegetable (e.g., soybean, sunflower) and animal (e.g., egg yolk) sources. LC has been commonly used in the food, pharmaceutical and cosmetic sectors, due to its recognized emulsifying properties along with its ability to be a viscosity modifier, wetting agent, dust-proof agent, etc. [87]. Generally, the commercially available “lecithin” or “lecithins” used in food applications actually represent a complex mixture of different phospholipids (such as phosphatidylcholine, phosphatidylethanolamine and phosphatidylinositol) and other lipids (such as TAG, glycolipids or sterols) [88].

##### Sources

The most commonly used forms of LC include those extracted from soybean, eggs, milk, sunflower, rice, etc. [88]. In relation to food BPs, LC represents another important by-product of the vegetable oil-refining process. Particularly, when vegetable oils are refined using a water degumming process, the BP obtained (wet gum) can represent a source of food-grade LC.

##### Application in OGs

This mixture of phospholipids is capable of forming spherical inverse micelles when used with a non-polar solvent (e.g., vegetable oils). Upon addition of a polar solvent (e.g., water), these micelles are stimulated to grow into tubular structures that self-assemble into a network capable of trapping the oil phase, resulting in the formation of OGs [24].

Bodennec et al. [49] carried out a characterization of LC-based OGs aimed at understanding the effect of water content on their microstructural organization. In the obtained OGs, the 3D network of inverted tubular micelles was held together by hydrogen bonds formed between the LC and water molecules and by hydrophobic interactions between the organic and aqueous phases. The results showed that a minimum LC concentration and an adequate molar ratio of water to LC played a key role in defining the structure and morphology of the final OGs. Below 7.5% LC, no gel formation was observed at any water content, confirming that a minimum LC concentration is required to induce gelation. Similarly, when the amount of water was insufficient for a given LC concentration, only a viscous solution (0.1 wt%) was obtained. In contrast, above a critical water concentration (>2 wt%), a clear macroscopic phase separation occurred, yielding a highly viscous phase and a non-viscous liquid phase. Under these conditions, water promoted LC precipitation, thereby preventing network formation. When 30 wt% LC was used, gelation occurred with 0.15–2.5 wt% water, remaining stable for 3 months.

Han et al. [51] studied different mass ratios between β-sitosterol and LC in the development of sunflower oil-based OGs, which were analyzed under three storage temperatures (5, 15 and 25 °C). This study revealed that the shape of the crystals constituting the network, as well as the rheological properties of the gels, varied significantly depending on the ratios between the two gelling agents and the storage temperature. When only one structurant was present, platelet-like crystals were formed, whereas when both structurants were used, the crystals’ shape was more needle-like. With higher storage temperatures (25 °C), the length of the microplatelets tended to increase, augmenting the crystal–crystal contact sites and consequently the resistance of the OGs obtained.

#### 3.1.5. Saponins

Previous studies have shown that, in general, the inclusion of synthetic and natural surfactants (e.g., glycerol monostearate (GMS), sorbitan monostearate (SMS), LC, mixtures of stearyl alcohol and stearic acid, etc.) in the formulation of OGs, along with the type and concentration of structuring agents and the type of solvent used, allow for modulating the physical characteristics (plasticity and hardness) of OGs and thus the sensory and texture properties of final products [89,90]. Davidovich-Pinhas et al. [91] highlighted that, beyond forming crystalline structures within the oil phase, these compounds may also interact with the polar regions of gelling agents (e.g., EC) owing to their amphiphilic nature. Such interactions can strengthen the oleogel network, resulting in OGs with enhanced mechanical strength and plasticity. In this context, plant saponins have emerged as promising food-grade surfactants. Quillaja saponin (QS), a triterpene from the soapbark tree (*Quillaja saponaria* Molina), has been approved for human consumption in many countries and organizations (World Health Organization, Food and Drug Administration, European Community, Food Standards Australia New Zealand, etc.), and is authorized as food additive E999 under European Regulation No. 1333/2008 [45,92]. QS has demonstrated the ability to create emulsions with a low surfactant-to-oil ratio (1:10, *w*/*w*), stabilizing them from variations in ionic strength, pH and temperature, through the union of high electrostatic and steric repulsion forces [93].

##### Sources

As mentioned above, there is an increasing need to replace synthetic food-grade surfactants with natural alternatives. Plant saponins are among the most promising candidates, particularly QS. The excellent emulsifying properties of quillaja extract have led to excessive exploitation of this exotic tree. Since saponins are amphiphilic secondary plant metabolites that are widely distributed in nature, an alternative source of them may be provided by agri-food BPs, which are currently available in abundance and still underutilized in higher-value applications. Relevant sources of saponins include legumes (e.g., peas, beans, soybean) and dicotyledonous plants (e.g., Hippocastani seeds, ginseng roots, quillaja bark, red beet, etc.), whereas most cereals are poor in saponins, with *Avena* representing a notable exception [94].

The processing of these products generates BPs (such as oat bran, beet husks, etc.) that can be used as a source of plant saponins with high surfactant activity, potentially suitable for OGs production and, at the same time, for the valorization of these side streams.

##### Application in OGs

However, to date there are still few studies aimed at characterizing the use of plant saponins, other than QS, in food production and specifically for the creation of OGs. One of the few available studies was carried out by Chen and Yang [92], who utilized QS as a unique stabilizer in emulsions to create orange oil powders and OGs through the emulsion-template approach. The results showed that this saponin is able to self-assemble into a fibrous network, forming a robust film at the oil–water interface. This structure stabilizes the emulsion and enables its use in oil powder production (via spray drying) and OG formation (via oven-drying), without structural collapse. Other available research focuses on the application of saponins for the creation of emulsions rather than OGs. However, studies on the emulsifying properties of saponins extracted from agri-food BPs provide a useful basis for evaluating their potential application in OG systems, particularly through indirect structuring approaches such as emulsion-template methods. Such strategies could also contribute to the valorization of these underutilized BPs. Some examples of particularly interesting studies are those performed on oats and red beet BPs:

• Oats (*Avena sativa* L.) are largely produced at the global level, with an annual production of more than 20 million tons [95]. One of the main BPs of oat milling is bran, which is considered a healthy ingredient due to the presence of β-glucans with a cholesterol-lowering effect [96]. Oats contain considerable amounts of triterpene saponins (such as monodesmosidic avenacin A1 and A2) and steroidal saponins (such as avenacosides A and B), which have demonstrated antitumor activity through several mechanisms, including inhibition of cancer cell growth by blocking the cell cycle and promoting apoptosis [97,98]. Ralla et al. [99] carried out a study to investigate whether the amphiphilic extract from oat bran could be used as a natural emulsifier. The surface activity of oat bran extract at the interfaces between hydrophobic and hydrophilic systems (oil–water and air–water, respectively), as well as the stability of oil–water emulsions, was investigated under diverse stress conditions. The results showed that oat bran extract acted as an ionic emulsifier, leading to the formation of negatively charged sub-micron emulsion droplets. The formed emulsions were stable over a wide pH range (4–9), upon heat treatment (50 °C), and during storage at 25 °C for up to 42 days.
• Red beet (*Beta vulgaris* ssp. *vulgaris* var. *conditiva*) is widely known for its deep dark red color attributed mainly to the cationic antioxidant betalain and is broadly used as a food colorant or additive [100]. Large quantities of red beet husks accumulate during industrial processing and are mainly used as animal feed. Amphiphilic extracts obtained from this hitherto underexploited plant secondary stream may represent a sustainable and innovative alternative to synthetic food additives and open the path for the creation of a new class of natural extracts derived from different vegetable sources. Ralla et al. [101] investigated the interfacial properties and emulsifying capacity of an extract obtained from red beet peels as an industrial BP in order to assess its potential to replace synthetically derived emulsifiers. The results showed that the extract contained high amounts of surface-active saponins that promoted the formation of small (approximately 1.36 μm), negatively charged droplets at a low emulsifier-to-oil ratio (0.75:10). The resulting emulsions remained stable over a wide range of environmental stresses, including variations in pH and temperature (<50 °C).

### 3.2. By-Products as Source of Antioxidants

Food BPs from tomatoes, potatoes and other horticultural products contain many compounds with antioxidant activity, such as phenolic compounds and carotenoids.

#### 3.2.1. Carotenoids from Tomato By-Products

The tomato (*Solanum lycopersicum*) production chain contributes to generating a large volume of BPs. The World Processing Tomato Council (WPTC) estimates that around 8 million tons of BPs are produced each year globally [102]. The solid residue that remains after the industrial processing of tomatoes consists of peels and seeds, which are rich in carotenoids, phenolic compounds, vitamins and dietary fiber. The main carotenoids present in tomatoes are lycopene (peel) and β-carotene (seeds) [103]. Many epidemiological findings support a link between the consumption of carotenoids, particularly lycopene, and a variety of health benefits, including the prevention of cardiovascular diseases [104]. Therefore, they also play a considerable role in human health, being significant dietary sources of vitamin A and acting as antioxidants by trapping reactive oxygen species (ROS), which helps reduce the oxidation phenomena of lipids, proteins and deoxyribonucleic acid (DNA) [105].

O’Sullivan et al. [106] made β-carotene (BC)-loaded OGs using CO and EC (as a gelling agent), and investigated the in vitro bioaccessibility and stability while assessing the bioactive delivery capacity of OGs. The findings highlighted that β-carotene did not have a negative impact on the formation of the EC network. Furthermore, the existence of the latter retarded the oxidation of BC compared to liquid oil, suggesting the ability of the polymer network to protect BC against oxidation, probably due to the fact that the oil and BC were contained in pockets within the network. This finding is interesting for extending the role of OGs as alternative approaches for the encapsulation of oxidation-sensitive species. The study showed that EC-OGs can be used to create slow- or timed-release food matrices for the delivery of fat-soluble compounds.

Cui et al. [107] investigated the effects of different concentrations of MAG (GMS) on the structure of OGs and on the solubility and chemical stability of β-carotene in OGs. In this study, β-carotene displayed greater solubility in GMS-OGs than in liquid oil, thus enhancing the solubility and chemical stability of BC. This result suggests the possibility of using OGs as a method to create β-carotene-enriched functional foods. Furthermore, the findings showed that the higher the content of GMS, the greater the strength of the gel network and the greater the retention ratio of β-carotene. Hence, OGs seem to be able to supply a larger amount of fat-active compounds with a high level of stability.

Dhulipalla et al. [108] produced an OG with coconut oil and stearic acid (as gelling agent), enriched with lycopene at different concentrations (0, 25, 50, 75, 100 wt% on 20 g oil). Both lycopene and stearic acid showed self-organizing crystal structures, without negatively interfering with the structuring mechanism. The results of the structure and oil-binding capacity analysis revealed the strong ability of these OGs to retain oil and lycopene within the 3D network. These findings demonstrate the possibility of preparing OGs with coconut oil and lycopene using specific combinations, thus resulting in efficient methods for the transport of this bioactive agent within foods.

#### 3.2.2. Phenolic Compounds from Potato By-Products

The potato (*Solanum tuberosum*) is a tuber representing the world’s fourth major agricultural product behind wheat, rice and corn [109]. A consequence of this high global demand is the generation of significant volumes of BPs, such as potato peels, which are rich in phenolic compounds [110]. These bioactives are associated with positive impacts on human health, because they protect the body’s cells from oxidative processes and show antioxidant, anti-inflammatory, antitumor, and anticancer properties. The polyphenols contained in potato peels comprise phenolic acids, flavonoids and anthocyanins. Beyond their antioxidant properties, potato peel extracts have been shown to possess antimicrobial activity, which further enhances their relevance as functional ingredients for food applications. This activity has been mainly attributed to the presence of phenolic compounds and other bioactive constituents of potato, including glycoalkaloids, whose biological properties have been comprehensively reviewed in studies addressing potato by-product valorization [109,110].

However, to date, no studies have explored the incorporation of potato by-product (BP)-derived extracts into OG formulations, and their antimicrobial activity within OG systems remains unexplored. Future investigations are therefore warranted to determine whether these extracts can enhance not only oxidative stability and bioactive compound delivery, but also the microbial stability of foods structured with OGs. Some research has been carried out on the use of certain phenolic compounds such as quercetin to formulate OGs.

Quercetin is a fat-soluble flavonoid found mainly in onions, tea, apples and berries, usually present in its glycosidic form; it is among the most abundant flavonoids in nature and one of the most studied polyphenols for its various biological activities [111,112].

Rocha-Amador et al. [113] prepared a quercetin-loaded OG using three different vegetable oils (canola, soybean and corn oil). The study revealed a higher bioaccessibility of the flavonoid in OGs made with highly glycosylated quercetin. The authors hypothesized that the presence of glycosides favors greater interaction with the soluble fraction, increasing bioaccessibility. The results revealed that OGs prepared with canola oil (CO) showed a greater level of crystallinity, a more stable needle network and greater bioaccessibility of quercetin. The authors suggested that a higher amount of MUFAs enhances intermolecular interactions, leading to a better-organized and stronger structure, which subsequently improves compound release. Therefore, in the production of these OGs, it is important to choose the type of oil according to the type of phenolic compound and its degree of glycosylation.

Recent studies have explored the use of both free and esterified phenolic compounds, such as cinnamic acid (a free phenol) and ethyl ferulate (an esterified phenol), in combination with PS to form bicomponent OG systems. Jia et al. [114] reported that when these compounds are combined with PS, they exhibit a synergistic effect on gelation, leading to the formation of stable and self-supporting gels. The crystal structures showed improved organization, and the rheological analysis showed higher G′ values and reversible gel behavior.

#### 3.2.3. Water-Soluble Polyphenols

Agri-food BPs, such as cereal brans and germs, grape peels and seeds, pomegranate peels and seeds, wine pomace extracts and flours, olive mill wastewater, olive pomace, and overripe berries, also represent valuable sources of polyphenols that can exert antioxidant activity [7,115,116]. In addition to their antioxidant properties, water-soluble polyphenols recovered from agri-food BPs may provide antimicrobial functionality, depending on their botanical origin and phenolic composition. This aspect is particularly relevant for by-products generated during olive oil production, such as olive mill wastewater and olive pomace, which are rich in hydrophilic phenolic compounds and have been associated with antimicrobial activity [7].

However, their antimicrobial effects have not yet been specifically investigated in OG systems. Therefore, future studies should assess whether these compounds can contribute to microbial stability when incorporated into OG-based or biphasic food systems.

Since many phenolic compounds present in these plant-derived by-products are hydrophilic rather than lipophilic, their incorporation into OGs poses additional challenges. Owing to the predominantly lipophilic nature of OGs, most studies conducted in this field have focused on the enrichment of OGs with fat-soluble bioactive compounds. Nevertheless, recent research has explored strategies for incorporating water-soluble compounds into OG formulations, thereby expanding the range of bioactive ingredients that can be delivered through these systems.

Shi et al. [117] created OGs enriched with tea polyphenols, well known for their antioxidant properties as free-radical scavengers. To overcome the problem of solubility in oil, the authors first prepared an emulsion of tea polyphenols–water–stearic acid–surfactant. This complex was then freeze-dried and used at different concentrations (1, 2, 4, or 6 g) in 20 g of peanut oil to create OGs by using a direct dispersion approach. The study showed that the antioxidant activity of OGs enriched with tea polyphenols was 2.5-fold higher than that of the synthetic antioxidants (butylated hydroxytoluene and propyl gallate) currently used in food formulations. This study provided an efficient approach to extend the formulation of OGs to water-soluble bioactive compounds.

Furthermore, OGs can be used as a basis to create other systems (OG-based emulsions, bigels, etc.), which have been found to be very efficient in transporting bioactive compounds of both lipo- and water-soluble natures. Among these, bigels are attracting great interest. They are hybrid gels that contain two immiscible liquid phases individually stabilized by gelling agents [9]. Such systems are generally created by the direct combination of OGs and hydrogels, subjected to stirring (about 600 rpm) at room temperature. Having both an oil and an aqueous phase, bigels may have the ability to simultaneously transport both lipo- and water-soluble compounds [118]. These hybrid systems are still rather unexplored, especially in terms of microstructural characteristics but, considering their great potential for pharmaceutical and food applications, it is likely to be further investigated in future years [119].

Among phenolic compounds, those derived from the extra virgin olive oil (EVO) chain are the best known. Indeed, olive oil phenolic compounds are currently the only phenolic compounds associated with an authorized health claim in the European Union. Commission Regulation (EU) No 432/2012 [120], which establishes a list of permitted health claims made on foods, recognizing their contribution to human health under specified conditions of use. The health claim in particular recognizes that “olive oil polyphenols contribute to the protection of blood lipids from oxidative stress”, which only applies to olive oil that contains at least 5 mg of hydroxytyrosol and its derivatives per 20 g of oil. Therefore, dietary intake of phenolic compounds from virgin olive oils significantly contributes to reducing the levels of oxidized low-density lipoprotein (LDL) in human plasma [121]. During the production of EVO, large quantities of solid waste and liquid effluents (olive pomace and vegetation waters) are generated. These BPs show a wide spectrum of antimicrobial activity, high acidity and phytotoxicity, which make their biological degradation and disposal problematic, constituting a problem in both economic and environmental terms. Usually, about 98% of phenolic compounds are found in BPs, while only a small percentage (about 2%) is transferred into olive oils during their production [122]. Hence, these BPs represent a rich source of phenolic compounds, which can be used as high-value ingredients for food and pharmaceutical applications. Most of the polyphenols commonly identified in olive mill vegetation waters (OVW) are phenyl alcohols, phenolic acids, secoiridoids and flavonoids [123], while in solid residues, secoiridoids represent the most abundant portion of total phenols, especially oleuropein and ligustroside [122]. Hydroxytyrosol (from the hydrolysis of oleuropein) is considered one of the highest-value compounds due to its high antioxidant activity and is currently used as a therapeutic agent, food supplement or natural ingredient in the food and feed industries [122]. These characteristics make phenolic compounds valuable ingredients to be used as natural antioxidants in meat product formulation [124,125]. Secoiridoid derivatives such as oleacein exhibit intermediate lipophilicity, due to their amphiphilic structure combining a polar catechol moiety with a more hydrophobic elenolic backbone. This dual character enables their partial solubility and partitioning into lipid phases, distinguishing them from both highly hydrophilic simple phenols and fully apolar lipid antioxidants [126].

Therefore, the use of OGs could be an alternative approach for incorporating and transporting bioactive compounds, potentially yielding many advantages in terms of protection against, for instance, oxidative phenomena in food systems [127]. Incorporation strategies usually allow to avoid the depletion of carried compounds during food storage, ensuring the retention and transport of an adequate amount of these compounds within food matrices [20]. The combination of these approaches would bring several advantages, both for the meat industry (in a perspective of progressive reduction in the use of synthetic antioxidants, such as BHT and nitrites) and for the olive oil industry, promoting a more sustainable approach to the management of its BPs.

### 3.3. By-Products as Network Reinforcing/Stabilizer Agents

#### 3.3.1. Fibers

In the formulation of OGs, it should be considered that the gel strength depends on several factors, including oleogelator type and concentration [128]. However, high gelator levels may affect the sensory quality of OGs and the final food product. Therefore, one of the main challenges lies in using reduced concentrations of structuring agents without compromising the mechanical properties of the gels. In this regard, dietary fibers are widely used in food formulations because of their ability to increase viscosity and consistency, improve water- and oil-holding capacity (WHC and OHC), stabilize high-fat systems, and provide technological functionalities such as thickening, emulsification, foaming, and gel formation [129,130]. In addition, their incorporation can enhance the nutritional profile of foods by increasing dietary fiber content. Numerous studies have demonstrated that dietary fibers also confer a range of health benefits, including a reduced risk of cardiovascular diseases, hypertension, diabetes, obesity, and various gastrointestinal disorders [128,131]. To date, both water-soluble fibers (pectins, β-glucans, galactomanan gums and a wide variety of non-digestible oligosaccharides, among them inulin) and insoluble fibers (cellulose, hemicellulose and lignin) are also largely employed as bulking agents for a partial replacement of other ingredients such as fat and flour [132]. Among insoluble dietary fibers, one of the main aspects influencing the ability to interact and retain water (WHC) is the high density of hydroxyl (-OH) groups characterizing the side chain of the cellulose skeleton. Although cellulose has numerous OH groups and a large hydrogen-bonding capacity, its chains exhibit amphiphilicity; however, hydrophobic interactions that could be exploited are often underestimated [133,134]. In particular, cellulose chains could participate in hydrophobic interactions with nonpolar molecules through -CH groups on glucose pyranoses [134]. In the case of emulsions, the interactions with both hydrophilic and hydrophobic phases may improve water and oil retention, physical and/or oxidative stability [132,135]. Therefore, this dual affinity may support their application in the formulation of OGs [128].

To date, most studies have focused on cellulose-derived materials such as EC, which remains the only known polysaccharide capable of structuring oils through a direct-dispersion approach. This ability is attributed to the presence of hydrophobic moieties within its molecular structure, which allow it to effectively gel hydrophobic liquids and form stable OGs [25]. In these systems, however, EC itself acts as the primary oleogelator. By contrast, the use of plant fibers or plant-derived materials as supporting or stabilizing elements within networks formed by other structuring agents remains largely underexplored, as does their impact on OG structure and stability. The ability of particles to modify mechanical properties by increasing the rigidity of food gels has been demonstrated in other systems. For instance, Gravelle et al. [136] showed that adding a 0.05 volume fraction of glass beads (4 μm) as a model hydrophilic filler to minced-meat gels increased hardness threefold. In contrast, the few studies investigating fibers or plant materials in OG production generally rely on indirect methods requiring prefunctionalization or high fiber loadings. David et al. [137] showed that insoluble cellulose fibers can structure rapeseed oil without thermal treatment; however, the powders must contain at least ≈60% cellulose, and dispersions prepared with shorter fibers require mass fractions close to ≈30 wt% to induce oleogelation. Such high levels may limit the applicability of this approach at an industrial scale or affect sensory acceptance. Among direct-dispersion approaches, Cheng et al. [40] proposed a novel method to prepare OGs by using only insoluble soy fibers (ISFs) and exploiting capillary suspension forces. OGs were produced by mixing oil and ISFs in a planetary ball mill to create a homogeneous suspension, followed by the addition of a controlled volume of water to induce capillary bridging. However, ISF volume fraction, oil polarity, and the properties of the secondary fluid (e.g., ionic strength, pH) strongly influence W/O interfacial tension and, consequently, the rheological and mechanical behavior of the resulting OGs, thus adding a further level of complexity.

To the best of our knowledge, the study by Principato et al. [128] is among the very few that have investigated the incorporation of plant fibers as reinforcing or stabilizing elements within a structuring network through direct addition to oil, without prior fiber functionalization. Nevertheless, further research is needed to elucidate how fiber addition into OGs or OG-based systems (e.g., bigels, emulsions) affects their mechanical, thermal, rheological, and stability properties, as well as to clarify the nature of possible chemical interactions in relation to fiber type and concentration, oleogelator characteristics, and oil composition.

##### Citrus Fibers

Citrus fruits are among the most widely grown fruit crops in the world, with an annual production of over 120 million tons and 110 million tons of citrus waste (citrus peels, pulp residues, and seeds) generated by the global citrus processing industry [138]. Citrus pomace is used extensively in the pectin extraction industry to optimize the use of citrus waste [139]. Citrus fiber seems to have a higher total content of dietary fiber and superior physico-chemical properties (such as WHC and water-swelling capacity (WSC)) compared to cereal fiber [129]. In addition to the many water-interacting hydroxyl groups present in the cellulose side chain, the adjacent cellulose chains create a stable strand-like arrangement by hydrogen bonds, which provides the cellulose of the citrus fiber with a good OHC [140]. Moreover, a recent work has shown that citrus fiber can act as the sole gelling agent in emulsion-templated oleogels, with network features that can be adjusted via enzymatic treatment [141]. The composition and content of dietary fiber are different among various kinds of citrus fruits and are related to the specific source and extraction method. Indeed, it should be noted that preparation procedures and/or the plant source from which the fiber is extracted, as well as intra-species differences (e.g., plant variety), significantly influence the functional properties of plant fibers. This aspect is clearly shown in the study by Barbut [142], who compared two citrus fibers differing in preparation procedures and found variations in their WHC when employed in meat systems.

He et al. [140] also studied the effects of composition (type and percentage of specific components) and microstructure on the WHC, water-swelling capacity (WSC) and OHC of nine citrus fiber samples; after removal of pectin, hemicellulose and lignin, the citrus fibers with cellulose as the main component showed the best OHC properties. This finding provides further insights and information for optimal fiber applications in OGs formulations. Phoon and Henry [134] developed a non-thermal method for producing OGs based exclusively on dietary fibers and vegetable oil, using either an emulsion- or foam-template approach. In their shear activation protocol, suspensions of insoluble dietary fibers in water were first subjected to high-shear homogenization to increase their surface activity. During homogenization, soluble fibers were added to promote their association with insoluble fiber chains, thus weakening their inter-chain hydrophobic interactions and facilitating their dispersion in water. Among the tested fibers, citrus fiber did not require co-solute addition, as it naturally contains both insoluble and soluble fractions. The resulting shear-activated fiber dispersions were then used to create fiber-based OGs via indirect routes (emulsion- and foam-template). The physical properties of the obtained gels were strongly influenced by the fiber network, particularly by the size of the fiber filaments (both initial and after homogenization). In general, OGs produced with longer fibers exhibited greater firmness and higher oil retention within the gel network, suggesting that oil entrapment is related to the extent of fiber wetting by the oil phase. Longer and/or more intricately arranged fiber filaments may provide a larger surface area for oil retention and physical entrapment within the network.

##### Tomato Fibers

As mentioned in the previous paragraph, tomato is one of the most widespread vegetables in the world. During the industrial processing of tomato, tomato pomace (TP) is obtained, which is a lignocellulosic BP composed of skin (27%), pulp (40%) and seeds (33%) [143]. According to García Herrera et al. [144], tomato skins have a considerable quantity of protein (14%) and high fiber values (41%), which are mainly constituted by insoluble portions. Tomato fibers bind together to form a reticular matrix that imparts structure to the product and retains free liquids, preventing them from escaping or separating. The presence of cellulose-based polysaccharides in the TP gives it a good ability to absorb or retain liquids [143] and, therefore, makes it an interesting ingredient for applications in OG formulation. Mustafa et al. [38] investigated the use of agri-food waste materials, specifically micronized tomato peel and coffee ground residues, to structure liquid oil via a capillary-bridge mechanism. The food residues were finely micronized (particle size < 10 µm) using high-pressure homogenization (70 MPa) and subsequently dispersed (25 vol%) in peanut oil. Capillary suspensions were obtained by adding distilled water as a secondary fluid, in amounts expressed as saturation ratios (or volume fractions of water to oil phase) ranging from 0.17 to 0.57. Because of the predominantly hydrophilic nature of the particle surfaces, the addition of water promoted the formation of capillary bridges between particles, resulting in the development of a three-dimensional network that entrapped the oil phase and yielded self-supporting OGs. The study demonstrated that finely micronized agri-food residues can effectively structure liquid oil through capillary-suspension principles, offering a clean-label alternative to conventional oleogelators while simultaneously valorizing food waste. Furthermore, the authors showed that adding a surfactant to the aqueous phase reduced the oil–water interfacial tension and substantially weakened the network structure, emphasizing the sensitivity of capillary gels to interfacial forces. Overall, this work provides a proof of concept for the sustainable development of oleogels from food processing BP, highlighting the pivotal role of interfacial tension in tailoring gel properties.

##### Bamboo Fibers

Bamboo fiber, obtained from the residues of bamboo processing, is gaining increasing attention due to its high dietary fiber content and functional properties. Bamboo is a plant belonging to the Poaceae family and is found worldwide in 1250 species belonging to 75 genera [145]. Bamboo species show impressive annual biomass growth, estimated at between 22 and 44 million tons per hectare per year [146], which consequently generates a high amount of bamboo BPs, especially shoots. The latter, owing to their rapid growth and maturation, short production cycle, and high nutritional value, are regarded as a sustainable nutritional resource that could contribute to meeting future food demand and enhancing food security as the global population continues to grow [147]. Bamboo shoots are rich in functional ingredients, including dietary fiber with cellulose, hemicellulose and lignin as the main carbohydrates present [148]. Bamboo shoot fiber is the most widely known product in the food industry. These fibers are commonly incorporated into bakery products, pasta, meat products, cheeses, and yogurts, increasing the dietary fiber content of the final products without significantly altering their color, odor, or flavor characteristics [145,149]. Moreover, when the relevant regulatory criteria are met, their inclusion may support fiber-related nutrition claims. For these reasons, bamboo fibers are also widely used as a fiber source in gluten-free products [150]. Due to its composition, bamboo fiber displays a high WHC, water-swelling capacity (WSC), and OHC [151,152]. Several studies have demonstrated the beneficial effects of bamboo fiber in food systems. For instance, the incorporation of bamboo fiber into bakery products leads to greater moisture retention and better texture qualities [153,154]. Similarly, its addition to meat products has been found to enhance emulsion stability and fat-binding capacity, thereby improving overall product quality [155,156,157]. The functional properties of bamboo fiber, as for all dietary fibers, vary considerably according to the plant source, extraction methods and processing conditions (degrees of purification, thermal treatment, etc.), chemical composition, structure, and granulometry [158]. Principato et al. [128] conducted one of the first studies aimed at investigating the effects of the direct addition of bamboo fiber on an OG system made of sunflower and extra virgin olive oils and RBW, as organic solvents and gelling agent, respectively. The results demonstrated that fibers, especially those with small particle size, can play a crucial role both as structuring agents and as stabilizers against temperature fluctuations related to the cooling rate during the network formation phase. In particular, 40 and 90 μm fibers showed a significant increase in structural properties, correlated to an increase in the fusion energy detected by differential scanning calorimetry (DSC) analysis, resulting in the formation of gels with higher resistance values. These findings highlight the potential of bamboo fiber as a functional ingredient for both food and OG formulations.

##### Brewer’s Spent Grain: A By-Product to Be Explored

Among the various BPs and wastes generated by the agri-food industry, BSG remains one of the most underutilized in OGs production, despite its promising composition and functional properties. BSG is the most abundant BP of the brewing industry, accounting for approximately 85% of the total solid residues generated and about 40% of overall beer manufacturing waste [159]. Globally, around 39 million tons of BSG are produced annually, with approximately 20 kg generated per hectoliter of beer brewed [160]. This BP is obtained after the malting and mashing steps, during which barley starch is converted into soluble sugars [161].

BSG primarily consists of the husks and insoluble fractions of barley grains and is particularly rich in dietary fiber, especially insoluble components (such as cellulose, hemicellulose, and lignin), as well as in proteins, phenolic compounds (e.g., ferulic and *p*-coumaric acids), and non-starch polysaccharides (such as β-glucans and arabinoxylan) [159,162,163]. This complex composition makes BSG a potential multifunctional ingredient for various food applications. Although traditionally used as animal feed or fertilizer due to its high moisture and spoilage risk, recent studies highlight its upcycling potential in functional foods, aligning with Sustainable Development Goal 12 [163]. BSG has already been explored as an ingredient in snacks, pasta, frankfurters, and bakery products, where it enhances fiber content and antioxidant activity [164]. Furthermore, its richness in β-glucans and fiber suggests significant potential as a functional ingredient with combined technological and health-promoting benefits. In the EU, authorized health claims state that oat and barley grain fiber contribute to an increase in fecal bulk; this claim applies only to foods that are “high in fiber” (≥6 g/100 g), as defined for nutrition claims in European Regulation No 1924/2006 [165]. EFSA also recognizes the cholesterol-lowering effects of β-glucans at a daily intake of 3 g, and their contribution, together with arabinoxylans, to reducing post-prandial glycemic responses [166,167,168]. In addition, several studies suggest that the arabinoxylan and β-glucan fractions contained in BSG may exert prebiotic effects by promoting the growth of beneficial gut microbiota, especially species belonging to the genera *Bifidobacterium* and *Lactobacillus* [169,170]. Moreover, Battistini et al. [171] demonstrated that incorporating BSG into fermented milk formulations enhanced the survival of probiotic strains when subjected to in vitro-simulated gastrointestinal conditions. More recently, Kruk et al. [172] showed that BSG can also serve as a suitable growth medium for probiotic bacteria. This potential prebiotic activity adds further value to the use of BSG in functional food formulations.

Recently, Chin et al. [161] investigated the potential of BSG as a plant-based emulsifier. Mild colloid milling produced particles smaller than 10 µm, with a composition of 26–32% protein and 52–62% fiber. In oil-in-water emulsions, the surface-active proteins from the soluble fraction reduced oil–water interfacial tension by approximately 35%. Emulsions stabilized with BSG fractions showed no signs of coalescence over 10 days of storage, even though the underlying stabilization mechanisms differed among fractions. Insoluble BSG particles promoted Pickering-type stabilization through interfacial bridging, whereas whole BSG exhibited a combined stabilizing effect arising from interactions between its soluble and insoluble components. Notably, when whole BSG was used, harsh extraction steps were avoided, supporting a more sustainable and clean-label approach. In another study, Choi et al. [173] evaluated the partial replacement of pork back fat with a BSG-based pre-emulsion in reduced-fat chicken sausages. When 20–25% of the pre-emulsion was incorporated, the fat content and caloric value were significantly reduced, leading to an improvement of texture parameters (hardness, gumminess, chewiness) without compromising sensory acceptance; these outcomes highlighted the technological and functional value of BSG-derived emulsions in reformulated meat products. Overall, given its unique composition and functional attributes, BSG represents a promising candidate to be used in lipid-based systems such as OGs, including in the development of healthier hybrid and plant-based food products. Its high insoluble fiber content may support network formation within gel matrices, while its phenolic compounds could enhance oxidative stability. Moreover, its low cost, wide availability, and nutritional value make BSG an attractive clean-label option for formulating sustainable and functional OGs. Nevertheless, its application in this specific context remains largely unexplored, requiring further research to fully elucidate its potential and optimize its performance in OG systems. Despite this potential, the application of BSG components in OG systems remains largely unexplored and should be addressed through targeted studies. Future research should clarify whether BSG is more suitable as a whole micronized ingredient, fiber-rich particle fraction, protein-containing interfacial stabilizer, or phenolic-rich antioxidant source. Key variables such as particle size, surface wettability, fiber/protein ratio, residual moisture and phenolic content should be correlated with oil retention, gel strength, rheological behavior, oxidative stability and sensory properties [161,163,173]. Capillary-suspension and emulsion-template approaches appear particularly promising, given the reported interfacial and Pickering-type stabilization properties of BSG fractions [38,161]. Further studies should also evaluate combinations with conventional oleogelators (such as waxes or lecithin), as well as assess stabilization, color/flavor impact and applicability in plant-based, hybrid, bakery or meat-reformulated products.

### 3.4. Cross-Sectional Comparison of BP-Derived Ingredient Classes in OG Systems

Based on the ingredient classes discussed above, Table 4 provides a cross-sectional comparison of BP-derived ingredients and related compounds for food OG formulation according to key criteria used in OG design, including OBC, gel strength, thermal behavior, sensory acceptability, scalability, and regulatory or food-grade readiness. This comparison also highlights that the suitability of each BP-derived ingredient class depends on the specific mechanism through which it contributes to oil structuring, stabilization, or functionalization. Moreover, from an application perspective, these ingredient classes differ markedly in terms of technological maturity and potential for industrial translation, which are strongly influenced by processing requirements, ease of incorporation into OG systems, sensory impact, availability, cost, and regulatory status.

Waxes primarily act as oil-structuring agents by forming continuous crystalline networks capable of immobilizing oil. Overall, waxes currently represent the most technologically mature BP-derived structuring agents because of their high OBC at relatively low concentrations and compatibility with direct (one-step) oleogelation approaches. In addition, wax-based OGs generally exhibit high oil retention, good gel strength, thermal stability, and tunable textural properties. However, their performance is strongly influenced by wax composition and concentration, oil type, wax–solvent interactions, cooling rate, and crystal morphology. At high concentrations, waxy mouthfeel or excessive firmness may also limit spreadability and compatibility with specific food matrices [41,42,43,44,46,47,56,58]. From a regulatory and application perspective, BW, CDW, and CRW exhibit higher food-grade readiness because of their established use as food additives, whereas less conventional BP-derived waxes (such as RBW, SFW, and sorghum waxes) may require source- and country-specific evaluations. Their application in real food systems appears particularly promising in bakery, frying, spreadable, and fat-replacement products, even though performance remains strongly dependent on both the food matrix and OG formulation [41,42,43,44,45,46,47].

PS also act as structuring agents through self-assembled fibrillar or tubular networks that can achieve high OBC when the sterol/co-gelator ratio, sterol solubility, and oil polarity favor co-assembly. Furthermore, PS-based systems are particularly attractive because they combine structure formation with potential bioactive functionality. However, their performance remains highly formulation-dependent; therefore, sterol crystallization, solubility, dosage, and bioavailability must be carefully considered [51,52,66,67,72,73,76,77,78]. From a regulatory perspective, PS-enriched foods are associated with specific health-claim requirements for blood cholesterol management, making dosage, physical state, bioavailability, and food category particularly relevant. Consequently, their performance in real food matrices should be evaluated not only in terms of structuring efficiency but also considering PS stability and bioaccessibility during processing, storage, and digestion [66,67,68,69,76,77,78].

From a regulatory and industrial perspective, LC also exhibits high practical readiness because of its established use as an emulsifier in food systems and its availability from oil-refining streams. Nevertheless, it is generally more effective as a surfactant or co-structurant than as a primary oleogelator. Unlike waxes and PS, its main contribution derives from interfacial organization, inverse micellar structures, and mixed co-structuring networks. Consequently, LC may improve physical stability and textural properties in emulsion-based or mixed OG systems, even though it is less reliable as a stand-alone oil-binding gelator. Its performance, influence on texture and physical stability, and applicability in real food matrices strongly depend on formulation variables, including phospholipid composition, water content, water-to-LC ratio, ingredient source, purity, and interactions with other gelators [49,50,51,85,86].

Protein- and fiber-based OG systems are highly relevant from both nutritional and sustainability perspectives, even though they generally require more complex processing routes, such as indirect and capillary-suspension approaches, respectively. Protein-based systems can provide high OBC, viscoelasticity, and nutritional value when porous, dried-template, foam-template, emulsion-template, or interfacial networks are successfully formed. However, rheological properties, texture, oxidative stability, and matrix compatibility are strongly influenced by protein source, denaturation and aggregation state, drying history, oil composition, and oil–protein interactions [53,54,82,83,84,85]. From a regulatory perspective, proteins obtained from established food co-products (such as whey or defatted oilseed meals) generally exhibit higher food-grade readiness than less conventional protein-rich BP fractions. Nevertheless, allergenicity, purity, processing history, and intended food use must still be carefully considered. Their performance in real food matrices should also be assessed on a case-by-case basis, as indirect processing and drying operations may affect oxidative stability and sensory quality.

Plant fibers and BP-derived particles may reinforce OGs by acting as scaffolds, fillers, or capillary-bridge-forming particles. Their effects on OBC, rheology, and texture depend largely on particle size, surface properties, wettability, residual moisture, and composition. In addition, compositional heterogeneity and sensory issues, including gritty or fibrous mouthfeel, excessive firmness, dryness, and color or flavor changes, remain important constraints affecting both consumer acceptance and matrix compatibility [36,38,126,132,134,137,161,163,173]. From both regulatory and application perspectives, plant fibers and fiber-rich BP can support clean-label and nutritionally improved formulations. However, their successful incorporation into food products depends on compositional standardization, particle-size control, moisture management, compliance with nutrition-claim requirements, and validation in the final food matrix. Their application appears particularly promising in plant-based, hybrid, bakery, and meat-reformulated products, even though performance remains strongly affected by ingredient variability and sensory acceptance [161,163,173].

Saponins should primarily be regarded as interfacial stabilizers, particularly in emulsion-template systems, where they contribute to physical stabilization and co-structuring rather than acting as primary oil-binding agents [88,90,97,99]. From both regulatory and sensory perspectives, their readiness remains highly source-dependent. Quillaja extract is currently the most established food-grade saponin source, whereas BP-derived saponin-rich extracts require further standardization of composition, purity, functionality, and sensory properties. Their performance in real food systems should be evaluated mainly in emulsion-based or mixed OG systems, where bitterness, astringency, foaming behavior, and interactions with other ingredients may influence both product stability and consumer acceptance.

Finally, phenolic compounds and carotenoids are particularly promising ingredients for OG functionalization and oxidative stabilization, even though they should primarily be regarded as bioactive ingredients rather than structuring agents. Depending on their chemical structure, polarity, concentration, hydroxylation or glycosylation pattern, and compatibility with the oil–gelator system, they may influence crystallization and network formation, exerting detrimental, neutral, or synergistic effects on gel microstructure, rheology, and overall stability [104,105,106,107,108,111,112,115]. From a regulatory and application perspective, their use depends on source, extraction process, purity, dosage, stability during processing and storage, and intended food category. Furthermore, color, flavor, solubility, partitioning behavior, degradation, and release from the OG matrix must be carefully considered, as these factors can strongly affect sensory quality, oxidative stability, and bioactive delivery in the final food product.

From a scalability perspective, commercial applications of oleogel-based lipid-structuring technologies remain limited. Most BP-based OG systems reported in the literature have only been investigated at laboratory scale, whereas pilot-scale validation and full industrial implementation remain scarce. To better assess industrial feasibility and scale-up potential, future studies should move beyond laboratory-scale formulation and include pilot-scale validation, testing in real food matrices, process reproducibility, ingredient standardization, cost assessment, and life cycle or techno-economic analyses. These evaluations are essential to determine whether BP-based OGs can provide meaningful industrial and sustainability advantages over conventional oleogelators and other solid-fat replacement strategies.

**Figure 6:**
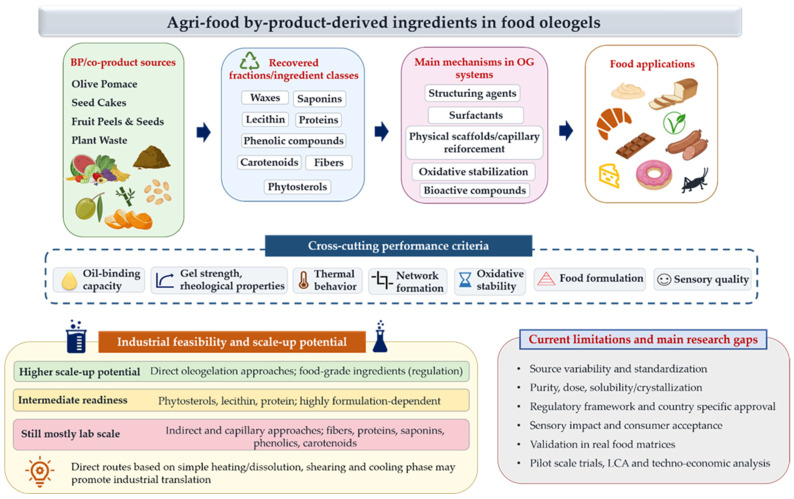
Conceptual overview of BP-derived ingredients in food OG: Sources, mechanisms, applications, feasibility, and current limitations.

Overall, the selection of BP-derived ingredients for OG formulation should be guided by both the underlying structuring mechanism and the characteristics of the target food matrix, considering not only the sustainable origin of the ingredient but also its structuring pathway, oil-binding behavior, oxidative effects, sensory impact, and compatibility with the intended food application. These relationships are summarized in Figure 6, which integrates BP sources, recoverable fractions, oleogelation mechanisms, food applications, industrial feasibility, and the main current limitations and research needs.

**Table 4:** Comparative assessment of BP-derived ingredients and related compounds for food OG formulation.

Ingredient Class	Main Role in OG Systems	Main Advantages, Structuring Ability and Thermal Properties	Functional Contribution	Main Constraints (Sensory, Scalability, Regulation)	Key References
**Waxes**	Structuring agents Crystalline network (usually thermo-reversible)	Can be used by direct approaches (one-step process, easier to scaled up). Widely available and relatively inexpensive (mainly when recovered as vegetable oil-refining side streams), with a heterogeneous composition resulting in a wide range of applications in OG formulation. High OBC (>90%), often at low concentrations (≥0.5 wt%); CGC greatly affected by wax type and concentration, crystal morphology and size, oil phase, and solvent–wax interactions. High melting points (61–85 °C), which depend on wax type and composition. Wax composition affects the melting point and crystallization kinetics, which in turn influence the thermal stability and the overall network strength, spreadability and mouthfeel.	Mainly contributes to network formation, thereby influencing oil retention, rheological, thermal and structural properties of OGs. Wax-based networks may also affect oxidative stability by acting as a “physical barrier”, thus reducing exposure to pro-oxidant factors (e.g., O_2_); however, the effect depends on wax type and composition, and final network structure.	Possible “waxy” flavor and mouthfeel, especially at high concentrations, with sensory impact highly wax- and matrix-dependent. Regulatory readiness is source- and country-dependent: beeswax (E901), candelilla wax (E902), and carnauba wax (E903) are well-established food-grade glazing agents under EU food additive regulation, whereas less conventional BP-derived waxes (such as rice bran, sunflower, or sorghum waxes), may require case-by-case evaluation depending on source, purity, intended food application, and country-specific regulation.	[41,42,43,44,46,47,56]
**Phytosterols**	Structuring agents and bioactive compounds Self-assembled fibrillar/tubular network Thermoreversible behavior under suitable formulation and processing conditions	Can be used by direct approaches (one-step process, easier to scaled up). Widespread in vegetable oils and recoverable from vegetable oil-refining side streams, especially deodorizer distillates Can form fibrillar/tubular networks. Performance depends on sterol ratio, oil polarity and minor oil components. Higher OBC (>90%) reached when a stable self-assembled network is formed, but limited by sterol solubility and crystallization. Thermal behavior affected by sterol crystallization, solubility and co-assembly.	High functional interest due to cholesterol-lowering effects Potential antioxidant contribution	Regarding the regulatory framework, plant sterols/stanols are associated with authorized EU health claims related to the maintenance or reduction of blood cholesterol levels under specific conditions of use. Therefore, dose, solubility, crystallization state, and bioavailability must be carefully evaluated in PS-enriched foods.	[50,66,67,72,73,76,77,112]
**Lecithin**	Surfactant, co-structurant, interfacial modifier Reverse micellar/tubular self-assembled network	Co-structurant or network modifier, but less universal as a stand-alone gelator. Can be used by direct approaches (one-step process, easier to scaled up). Oil immobilization is mainly associated with inverse micellar or mixed co-structuring networks, and effective gelation generally requires water or combination with other gelators. Thermal behavior is mainly governed by the stability of inverse micellar or mixed co-structuring networks, which depends on phospholipid composition, water/lecithin ratio and interactions with other gelators.	Can improve physical stability by promoting interfacial organization and stabilizing emulsion-based or mixed OG systems. Its functional contribution depends on lecithin source, purity, phospholipid profile, and interactions with water or other gelators.	High food-grade readiness, as lecithins are widely used food emulsifiers and are authorized in the EU as food additive E322. However, performance depends on source, purity, phospholipid profile, and intended food application.	[43,47,48,49,85,86]
**Proteins**	Structuring agents mainly through indirect approaches Polymeric networks (not thermo-reversible)	Used in oleogelation through indirect approaches. Positive effects on nutritional value and consumer perception. OBC can be high (>80–90%) when porous, interfacial or dried-template networks are properly formed. Thermal behavior depends more on protein network formation and processing history than on lipid crystallization alone.	Proteins may add nutritional value and can provide relevant technological functionalities (e.g., emulsification, foaming, interfacial stabilization, water/oil retention, and network formation). The effects on oxidative stability may vary according to protein source, denaturation/aggregation state, oil composition, and oil–protein interactions.	Require indirect approaches (multi-step processes); thus, the scalability is not as straightforward as the direct approach. Processing complexity and scalability remain limitations.	[53,54,82,83,84,85]
**Saponins**	Natural surfactants, interfacial modifier	Mainly relevant as interfacial stabilizers rather than direct oil structuring agents. They can support OG formation through emulsion-template routes and, in combination with other gelators, may contribute to co-structuring or network modification. Thermal behavior is mainly linked to emulsion/interfacial stability during processing and drying.	May improve physical stability mainly through interfacial stabilization. Depending on the botanical source and composition, saponin-rich extracts may also provide additional bioactive functionality.	Regulatory and sensory readiness is source-dependent. Quillaja extract is the most established food-grade saponin source, whereas BP-derived saponin-rich extracts require standardization of composition, purity and functionality, together with sensory assessment.	[88,90,97,99]
**Carotenoids and phenolic compounds**	Antioxidants and bioactive compounds	Usually not primary oil-binding or structuring agents. Their incorporation depends on solubility, partitioning behavior, crystallization tendency, and compatibility with the oil–gelator system. In some cases, they may modify gelator crystallization, network organization and thus gel stability.	Strong potential for improving oxidative stability, bioactive delivery and functional value of OG-based foods.	Possible color, flavor, solubility/partitioning, possible crystallization interference and degradation issues. Regulatory readiness depends on source, extraction process, purity, use level and intended food application.	[104,105,106,111,112,115]
**Plant fibers**	Stabilizers, fillers/scaffolds or capillary-suspension/bridging structuring agents	Promising for network reinforcement or capillary-suspension structuring. An indirect effect on thermal behavior may arise from network reinforcement, possible changes in crystallization behavior, and/or water-mediated structuring.	May improve physical stability, structural properties and OBC by acting as fillers, scaffolds, or capillary-bridge-forming particles. Can support clean-label and nutritionally improved formulations and, when EU nutrition-claim thresholds are met in the final product, may contribute to “source of fiber” or “high fiber” claims. Antioxidant contribution depends on the botanical source, fiber composition, and potential residual bioactive compounds.	High sustainability and clean-label potential, but variability linked to botanical origins, heterogeneous composition (especially for fibrous BP not subjected to fiber extraction), pre-treatments, particle-size distributions and sensory effects (e.g., fibrous/gritty mouthfeel, excessive firmness/dryness, possible color or flavor changes), strongly challenge their application in OG. Performance variability depends on particle size, surface properties, fiber composition and potential water content within the system.	[36,38,126,132,134,137,163,173]

Footnotes: BP: By-product; CGC: critical gelling concentration; OBC: oil-binding capacity; OG: oleogel; PS: phytosterol.

## 4. Use of Oleogels Formulated with Agri-Food By-Products for Food Applications and New Frontiers

OGs have been investigated for a wide range of food applications, including bakery products, meat and processed meat, dairy and cheese-type products, confectionery, fried foods, and spreads. The existing literature highlights the remarkable versatility of OGs as food structuring systems. However, the performance of BP-based OGs in food applications is highly formulation-dependent, with the final technological outcome being influenced by multiple factors, including oil type, oleogelator nature and concentration, and the structuring approach adopted [26,42,47,58]. When included in food, these OG variables will further interact with the matrix components, thereby affecting texture, microstructure, and sensory performance [174,175,176]. In this context, the incorporation of agri-food BPs and BP-derived ingredients may further expand the technological and sustainability potential of OGs, enabling the development of more tailored and functional formulations and opening new opportunities for innovative food systems, including plant-based and hybrid products. Moreover, OGs represent a valuable system to upcycle insect co-products and BPs.

### 4.1. New Frontiers

#### 4.1.1. Plant-Based and Hybrid Products

The use of OGs in plant-based and hybrid food products is still a partially explored field, representing a promising area for future research. The British Standards Institution (BSI) introduced PAS 224:2020 [177], the first specification defining “100% plant-based” foods as those whose key ingredients and processing aids are exclusively of plant origin. Subsequently, the International Organization for Standardization (ISO) issued ISO 8700:2025 [178], establishing an international framework for consistent terminology and labeling. Conversely, hybrid foods combine animal- and plant-derived ingredients to partially replace the animal fraction, improving nutritional and sustainability profiles while retaining familiar sensory attributes [179,180,181,182]. A key technological challenge in both categories is the replacement of animal fats, as current formulations often rely either on tropical oils rich in SFAs (e.g., palm and coconut oils), which provide solid-like textural properties but raise nutritional concerns, or on PUFA-rich liquid oils (e.g., canola oil), which improve the FA profile but are more susceptible to oxidation and may lead to the development of rancid off-flavors [183,184,185,186]. OGs represent a promising alternative, as they can provide functional properties similar to those of solid fats while potentially improving the nutritional profile of these products [187]. At the same time, increasing consumer awareness of the relationship between diet and health has stimulated the development of foods with improved nutritional profiles, often through fortification with bioactive compounds that typically exhibit poor dispersibility in aqueous matrices and low bioavailability. In this context, bioactive-loaded OGs formulated with agri-food BP-derived ingredients could help mitigating oxidative damage to lipids and proteins during processing and storage, while simultaneously enhancing the dispersibility and bioavailability of incorporated bioactives by protecting them against oxidation and loss of functionality [9]. Moreover, OGs offer the possibility to design the microstructure and rheological behavior of the fat phase, tailoring it to specific plant-based or hybrid matrices. Although the literature on OGs in plant-based and hybrid foods is still limited, some recent works highlight their potential. Moon et al. [188] replaced palm oil in a plant-based cheese analog with an OG based on canola oil and CRW, achieving a marked reduction in the SFA/UFA ratio and an improvement in elastic properties. Ropciuc et al. [189] developed plant-based ice creams with OGs formulated with hemp seed and olive oils structured with CDW (3–9%), which showed increased hardness, oil retention and oxidative stability at higher wax levels. In plant-based cheeses, Fidan et al. [190] structured apricot kernel oil with CRW/CDW blends to replace coconut oil, drastically reducing SFA content while maintaining suitable melting behavior. Noon et al. [191] replaced coconut oil with PS-based OGs in vegan cheese, reducing SFAs from 28% to 2% and improving meltability while keeping texture highly tailorable.

Overall, current evidence suggests that OGs can help reconcile technological performance, nutritional improvement and clean-label formulation in plant-based and hybrid foods. Future work should clarify how OG structure interacts with complex plant-based matrices, and how BP-derived ingredients can be strategically incorporated within OGs to control oxidation, confer adequate structural properties, and deliver bioactive compounds.

#### 4.1.2. OGs as a Way to Valorize Insects by- and Co-Products

Driven by population growth and the need for more sustainable protein sources, edible insects are increasingly considered valuable food ingredients [192]. Several species have been authorized in the EU, including *Tenebrio molitor*, *Locusta migratoria*, *Acheta domesticus* and *Alphitobius diaperinus* (Commission Implementing Regulation (EU) [193,194,195,196]. Beyond proteins, insect fat (≈10–50% dry basis) represents a promising co-product [192,197], whose FA profile varies with species, sex, metamorphic stage, diet, environmental temperature, migratory flight and can also be affected by extraction methods [198,199]. Insect oils are often rich in UFAs, mainly linoleic (C18:2 *cis*-9,12) and oleic (C18:1 *cis*-9) acids, and in some species (e.g., crickets) can also contain long-chain *n*-3 PUFA [200,201,202]. This composition makes them promising candidates as liquid phases for OGs, even though only few studies have examined this application. Kim and Oh [203] structured *T. molitor* oil with CDW, CRW and BW, improving its oxidative stability; CRW-based OGs also showed high viscoelasticity and melting point, performing as suitable shortening replacers in cookies. Jeong and Oh [204] confirmed that BW/GMS blends (3:1) can strengthen TM-oil OGs, increasing melting point and viscoelasticity as the percentage of BW rises and with GMS addition. Overall, the application of insect oil in OG formulation remains an emerging area but shows strong potential for integrated fat structuring with co-product valorization and upcycling. Further research is needed on formulation optimization and consumer acceptance. In addition to technological optimization, the use of insect-derived oils and co-products in OG-based foods requires careful consideration of food-safety and regulatory aspects, including species authorization under novel food regulations, extraction residues, microbiological and chemical contaminants, allergenic potential, oxidative stability, and labeling requirements [192,193,194,195,196,197,198,199,200,201,202].

## 5. Research Gaps and Future Perspectives

Despite the promising potential, several research gaps must still be addressed before BPs and their derived ingredients can be widely employed in OG systems and, subsequently, in food matrices:

(i) First, oleogelation itself is still a relatively recent technology, and there is a need for a better understanding of how a given oleogelator behaves in different oils and in the presence of BP-derived components. The same structuring agent may display markedly different solubility, critical gelling concentration, and network morphology depending not only on the FA composition of the oil, but also on the presence and level of minor polar components. These factors modulate oleogelator–oil interactions and, consequently, the formation and properties of the 3D network [55,76,205].
(ii) When BPs or BP-derived compounds (e.g., phenolics, carotenoids, and saponins) are incorporated into OG systems, they may further modify the local chemical environment around gelators and interfacial regions, thereby influencing crystallization, network microstructure, OBC, and overall physical stability in either a beneficial, neutral, or detrimental manner [92,114,206,207]. Based on the available literature, no clear evidence currently supports the use of a single chemical descriptor (such as hydroxyl group density) to predict detrimental interference with wax-based 3D networks. The available evidence suggests that the outcome is highly formulation-dependent, being influenced by the specific oil–wax system, minor oil components, and the compatibility between the added bioactive compounds and the oleogelator network [55,58,114]. This is particularly relevant when BPs are used directly, without extraction or purification of specific compounds, since their multicomponent nature introduces additional functional groups and minor constituents that may influence network formation in either a beneficial or detrimental manner. It is therefore essential to evaluate how their incorporation affects the 3D network formed by a given oleogelator (or oleogelator blends). In this perspective, future research should combine systematic formulation studies with rheological, thermal, microstructural and spectroscopic analyses. A detailed characterization of BP-derived ingredients is also needed to clarify how they affect oleogelator–oil interactions and network formation. Factorial experimental designs and advanced statistical methodologies (such as response surface methodology) will therefore be required to systematically disentangle the individual effects of oleogelator type and concentration (single or mixed), BP type, and level of inclusion, thereby supporting the optimization of BP-based OG formulations.
(iii) Furthermore, crude BPs often exhibit heterogeneous and irregular particle size distributions, along with complex surface properties, which can significantly influence oil wettability, adsorption behavior and, consequently, OBC and structural integrity of gels [38,208]. Future studies should clarify how the composition and structure of these BP-derived ingredients determine their function and interactions with different oleogelators and oils and evaluate their performance in complex food matrices beyond model systems.
(iv) Another critical aspect arises from the complexity of BP ingredients that do not dissolve in oil, such as plant fibers from commercial ingredients or minimally processed BPs (e.g., BSG). Despite being often introduced as inert fillers or scaffolds, their presence can still influence the structuring process in ways that are not yet fully understood, and this remains a key area requiring deeper investigation [38,134,209]. BP-derived fibers can vary considerably in botanical origin, composition (e.g., soluble and insoluble fractions, pectin and phenolic content), particle size, and concentration; consequently, their interactions within oleogel networks, often assumed to be predominantly physical, remain poorly characterized, particularly when combined with different types of oleogelators, especially those bearing polar functionalities. As a result, the same BP-derived fiber may affect network formation differently depending on the structuring agent (e.g., waxes vs. MAGs). Particle size, density, surface porosity and roughness become particularly critical for BPs that do not dissolve in the oil phase, since excessively large, poorly wetted or highly irregular particles may disrupt network continuity and compromise physical stability [38]. Therefore, the different composition, shape, method of incorporation and possible pre-treatments of BPs, can strongly affect both BP–oleogelator and oleogelator–oil interactions, thus leading to gels with different rheological and thermal properties, microstructure, oil retention, and physical stability. Considering all these factors, future studies should systematically vary BP physical parameters (e.g., particle size distribution and surface properties) and correlate them with OG microstructure and performance, with the aim of identifying processing conditions that enhance oil structuring while maintaining the desired texture and sensory profile of the final product.
(v) Finally, regulatory issues and consumer acceptance must also be addressed to facilitate the transition of BP-based oleogels from laboratory development to industrial scale. From a consumer-acceptance standpoint, terminology should also be carefully considered, since “waste” or generic “by-product” wording may carry negative connotations. When scientifically and legally appropriate, more specific terms such as “upcycled”, “recovered”, or “sustainable plant-derived ingredient” may help communicating valorization without misleading consumers.

Overall, these research efforts will be pivotal to fully unlock the potential of BP-based OGs as tools for nutritional improvement, BP valorization and sustainable innovation in the agri-food sector.

## 6. Conclusions

Nowadays, the food industry faces an increasing demand for products with improved nutritional profiles, lower environmental impact, and adequate sensory quality. Within this framework, the replacement of traditional solid fats remains one of the major technological challenges. OGs obtained through direct or indirect structuring approaches represent a promising solution, as they enable the design of lipid systems that mimic the technological functionality of hard fats while preserving the favorable fatty acid profile of mono- and polyunsaturated vegetable oils. At the same time, the large quantities of food by-products (BPs) generated throughout agri-food supply chains highlight the need for effective valorization strategies capable of improving both environmental and economic sustainability.

This review demonstrates that BPs and BP-derived ingredients can contribute to OG formulation through multiple mechanisms, acting as structuring agents, stabilizers, fillers, surfactants, antioxidants, and sources of bioactive compounds. Their multifunctionality offers opportunities to simultaneously improve the nutritional, technological, and sustainability performance of food products. Particularly promising applications include plant-based and hybrid foods, where BP-based OGs may help reproduce the technofunctional and sensory roles of animal fats while improving fatty acid composition and enabling the incorporation of natural bioactive compounds. Among the BPs discussed, brewer’s spent grain (BSG), plant fibers, olive mill by-products, and insect co-products emerge as especially interesting multifunctional ingredients.

However, the current body of evidence also highlights important limitations that prevent the widespread adoption of BP-based OGs. Most studies remain confined to laboratory-scale model systems, whereas validation in real food matrices is still limited. Furthermore, the effects of BP compositional variability, particle characteristics, source-dependent functionality, and interactions with oil–gelator networks remain poorly understood. As a result, the performance of BP-derived ingredients cannot yet be reliably predicted across different oils, oleogelators, and food applications. Additional challenges include ingredient standardization, sensory quality, regulatory readiness, scalability, and industrial feasibility.

Future research should therefore move beyond proof-of-concept studies and focus on a set of clearly defined priorities: (i) compositional standardization and detailed characterization of BP-derived ingredients and fractions; (ii) mechanism-driven formulation studies linking source, purity, dose, particle properties, oil–gelator interactions, crystallization behavior, OBC, rheology, oxidative stability, and sensory quality; (iii) validation in real food products under realistic processing and storage conditions; (iv) pilot-scale studies addressing process reproducibility, ingredient availability, scalability, and cost-effectiveness; and (v) integrated life cycle assessment, techno-economic analysis, regulatory evaluation, sensory and consumer acceptance studies, and nutritional validation.

The future success of BP-based OGs should not be assessed solely on the basis of their ability to structure oils or improve fatty acid profiles. Their true value will depend on whether they can provide measurable advantages at industrial scale while maintaining product quality, consumer acceptance, economic feasibility, and environmental sustainability. Moreover, the nutritional implications of BP-based OGs remain insufficiently explored. OG structure, oleogelator type, network strength, and interactions with food matrices may influence lipid digestion, bioactive release and absorption, satiety responses, and gastrointestinal functionality. Since *in vivo* evidence remains scarce, these aspects should become a priority for future research.

Overall, the transition of BP-based OGs from promising laboratory concepts to viable industrial solutions will require a multidisciplinary effort integrating food chemistry, colloid science, process engineering, sensory science, nutrition, sustainability assessment, and regulatory research. Addressing these challenges will be essential to fully exploit the potential of BP-based OGs as tools for fat reformulation, by-product valorization, and the development of more sustainable food systems.

## Figures and Tables

**Table 1:** Classification of oleogelators based on molecular weight.

Oleogelators		
Molecular Weight	Potentially Recoverable from BP/Co-Products	Conventional Non-BP-Derived
LMW	Waxes (e.g., RBW, SFW, SGW), phytosterols (PSs), lecithin (LC), fatty acids (FA)	Fatty alcohols (FAOHs), monoacylglycerols (MAGs)
HMW	Protein-based (e.g., wheat protein, soybean protein, β-lactoglobulin, caseinate)	Cellulose derivatives such as ethyl-, methyl-, hydroxypropyl-cellulose (EC, MC, HPMC), xanthan gum

**Table 3:** Melting points of some of the main waxes used for OG formulation [43,59,60,61].

Wax	Melting Point Range (°C)
Beeswax	61–65 °C
Carnauba wax	80–85 °C
Candelilla wax	68–73 °C
Rice bran wax	75–82 °C
Sorghum wax	77–85 °C
Sunflower wax	75–80 °C

## Data Availability

Data sharing is not applicable to this article as no new data were created or analyzed in this study.

## Abbreviations

The following abbreviations are used in this manuscript:

BP	By-product
BSG	Brewer’s spent grain
BW	Beeswax
CDW	Candelilla wax
CGC	Critical gelling concentration
CRW	Carnauba wax
EC	Ethylcellulose
FA	Fatty acid
FAOH	Fatty alcohol
HMW	High molecular weight
LC	Lecithin
LMW	Low molecular weight
MAG	Monoacylglycerols
MUFA	Monounsaturated fatty acid
OBC	Oil-binding capacity
OG(s)	Oleogel(s)
PS	Phytosterol
PUFA	Polyunsaturated fatty acid
QS	Quillaja saponin
RBW	Rice bran wax
SFA	Saturated fatty acid
SFW	Sunflower wax
SGW	Sorghum wax
SPI	Soybean protein isolate
TFA	*Trans* fatty acids
WPI	Whey protein isolate
